# Whole-Genome Phylodynamic Analysis of Respiratory Syncytial Virus—Maryland, USA, 2018–2024

**DOI:** 10.3390/v18030331

**Published:** 2026-03-07

**Authors:** Ting-Xuan Zhuang, Amary Fall, Julie M. Norton, Omar Abdullah, Andrew Pekosz, Eili Klein, Heba H. Mostafa

**Affiliations:** 1 Johns Hopkins School of Medicine, Department of Pathology, Division of Medical Microbiology, Meyer B-121F, 600 N. Wolfe St., Baltimore, MD 21287, USA; tzhuang2@jh.edu (T.-X.Z.);; 2 W. Harry Feinstone Department of Molecular Microbiology and Immunology, The Johns Hopkins Bloomberg School of Public Health, Baltimore, MD 21287, USA; 3 Department of Emergency Medicine, Johns Hopkins School of Medicine, Baltimore, MD 21287, USA; 4 Center for Disease Dynamics, Economics, and Policy, Washington, DC 20005, USA

**Keywords:** respiratory syncytial virus, phylodynamic, RSV, evolutionary dynamics, genetic diversity

## Abstract

Respiratory syncytial virus (RSV) is a leading cause of respiratory infections in infants and older adults, with epidemiological patterns shaped by viral evolution and diversity. To investigate the molecular epidemiology of RSV before and after the COVID-19 pandemic, we conducted genomic surveillance and phylodynamic analyses of RSV-A and RSV-B circulating in Maryland from 2018 to 2024. Whole-genome sequencing of RSV-positive samples (n = 451) was performed, and genomes were analyzed with phylogenetic and Bayesian methods to estimate evolutionary rates, population dynamics, selection pressures, and genetic diversity. RSV-A predominated in most seasons, while RSV-B showed episodic surges in 2018 and 2023. All RSV-A genomes belonged to the ON1 genotype, and RSV-B belonged to BA9, with sequential clade dominances including A.D.1, A.D.5.2, A.D.1.6, and B.D.E.1 across different epidemic seasons in Maryland. Bayesian analyses estimated evolutionary rates of 7.07 × 10^−4^ substitutions/site/year for RSV-A and 1.02 × 10^−3^ substitutions/site/year for RSV-B and temporal fluctuations in effective population size linked to pandemic-related disruptions. RSV-A displayed greater overall entropy, yet RSV-B evolved slightly faster. Genetic variability was concentrated in the G glycoprotein, with positively selected sites at codon 273 (RSV-A) and codon 217 (RSV-B). These findings demonstrate temporal fluctuations in RSV-A and RSV-B predominance, clade replacement, and ongoing viral adaptation throughout the COVID-19 era, underscoring the importance of integrated genomic and phylodynamic studies.

## 1. Introduction

Respiratory syncytial virus (RSV) is a major cause of acute respiratory tract infections globally, leading to significant morbidity and mortality among infants, immunocompromised individuals, and older adults [1]. As an RNA virus characterized by rapid evolutionary dynamics and high genetic variability, RSV is classified into two distinct antigenic groups, RSV-A and RSV-B, each with multiple circulating genotypes [2]. RSV genotypes, historically defined by G gene variation (14 for RSV-A and 37 for RSV-B), were later reshaped by the emergence of the BA9 (RSV-B) and ON1 (RSV-A) genotypes, which rapidly displaced earlier genotypes and became globally dominated [3]. More recently RSV was classified into 3 RSV-A and 7 RSV-B genotypes, with whole-genome analyses resolving 24 RSV-A and 16 RSV-B clades [4,5].

Mutations drive the molecular evolution of RNA viruses, influencing their genetic variability, adaptability, and overall evolutionary trajectory [6]. RSV exhibits continuous evolutionary change driven by selective pressure from host immunity and antiviral interventions [7,8]. This dynamic evolution manifests genetically through nucleotide substitutions and amino acid alterations, affecting infectivity, virulence, and immune evasion [9].

Previous studies have provided valuable insights into RSV prevalence, strain circulation, and phylogenetic relationships across various seasons through genomic surveillance efforts [10,11,12]. However, phylodynamic analyses that integrate evolutionary, temporal, and demographic information to understand the transmission dynamics remain limited, especially in the context of changing epidemiological patterns such as those observed during and after the COVID-19 pandemic. Detailed investigations into RSV’s evolutionary dynamics across diverse geographic and temporal contexts are critical, as such insights inform public health interventions and guide vaccine development strategies.

In this study, we conducted a retrospective phylogenetic and phylodynamic analysis using longitudinal genomic data collected from the Johns Hopkins Health System (JHHS) over seven consecutive years, from 2018 to 2024. Our aim was to understand the genetic diversity, evolutionary patterns, and phylogenetic relationships among RSV strains circulating locally in Maryland pre, during, and post the COVID-19 pandemic.

## 2. Materials and Methods

### 2.1. Patient Consent and Study Specimens

The study received approval from the Johns Hopkins Institutional Review Board (IRB00221396, initial approval on 29 October 2019) and was granted a waiver of consent. Respiratory specimens were collected retrospectively with collection dates from 2018 to 2024. Standard-of-care RSV diagnostic testing at JHHS during this period was performed using the Cepheid Xpert Xpress SARS-CoV-2/Flu/RSV, the Xpert Xpress Flu/RSV, the NxTAG-RPP, or the cobas ePlex RP1/RP2 respiratory panels [13,14].

### 2.2. RNA Extraction, Real-Time RT-PCR Assay, and Two-Step Multiplex RT-PCR

RNA was extracted from 300 μL of clinical specimen and eluted in 60 μL volume using the Chemagic Viral DNA/RNA 360 Kit (Revvity, Waltham, MA, USA). Cycle threshold (Ct) values were determined via a research-use-only RT-qPCR assay targeting the RSV matrix (M) gene, utilizing the Luna^®^ Universal Probe One-Step RT-qPCR Kit (E3006) from New England Biolabs (NEB) (New England Biolabs, Ipswich, MA, USA) [15]. First-strand cDNA synthesis was carried out using the LunaScript RT SuperMix Kit (NEB) with 8 μL of extracted RNA in a total reaction volume of 10 μL under the following conditions: 25 °C for 2 min, 55 °C for 10 min, and 95 °C for 1 min. Specimens with Ct ≤ 35 were selected for downstream sequencing. Amplification of cDNA was performed in two multiplex PCR reactions (Pool 1 and Pool 2). Each PCR reaction (25 μL) contained 5 μL of cDNA, 12.5 μL Q5 Hot Start High-Fidelity 2× Master Mix (NEB), 3.5 μL nuclease-free water, and multiplex primers at final concentrations of either 200 nM or 400 nM (achieved by adding 0.5 μL or 1.0 μL of each 10 μM primer stock, respectively). PCR conditions included initial denaturation at 98 °C for 30 s, followed by 40 cycles at 98 °C for 10 s, 50 °C for 30 s, 72 °C for 4 min, and a final extension at 72 °C for 10 min. Primer sequences are listed in Supplementary Table S1.

### 2.3. Library Preparation, Genome Assembly, and Phylogenetic Trees Construction

Sequencing libraries were prepared using the NEBNext ARTIC Library Prep Kit and the Oxford Nanopore Native Barcoding Kit 96 (V14). Libraries were sequenced using an Oxford Nanopore GridION instrument (Oxford Nanopore Technologies, Oxford, UK). Sequence data were processed with an established in-house bioinformatics pipeline, as previously described [16]. Closest reference genomes were identified by BLAST (2.17.0) (MH447951 for RSV-A and OP975389 for RSV-B), and draft genomes were assembled with the mini_assemble module in Pomoxis. Consensus polishing was performed with Medaka, and sequencing depth was assessed using samtools. Quality control filters were applied to retain sequences with mean quality scores ≥ 30, and low-quality or incomplete genomes were manually excluded after sequencing. RSV sequences were assigned to clades using Nextclade (v3.19.0), and multiple sequence alignment was conducted using MAFFT v7 [17,18]. Phylogenetic trees were constructed employing maximum likelihood methods implemented in IQ-TREE v2.4.0, using 1000 bootstrap replicates. ModelFinder in IQ-TREE identified GTR + F + I + R3 as the optimal nucleotide substitution model [19]. Temporal signals were assessed using a root-to-tip regression in Tempest v1.5.3 with the best-fitting root [20]. Tree visualization was performed with FigTree v1.4.4. Additional reference sequences were used only in phylogenetic analyses (Supplementary Table S2) but were excluded from Bayesian analyses.

### 2.4. Estimated Evolutionary Rates, Bayesian Skyline Plot, and MCMC Trees Reconstruction

RSV evolutionary dynamics were investigated using Bayesian Markov Chain Monte Carlo (MCMC) analyses in BEAST v2.7.7 [21]. Of the four clock models tested (strict, uncorrelated exponential, uncorrelated lognormal, and random local), the uncorrelated lognormal relaxed clock provided the best fit with the highest log marginal likelihood (Supplementary Table S3). Bayesian Skyline Plot (BSP) analyses utilized the GTR + Γ substitution model, an uncorrelated lognormal relaxed molecular clock, and a Bayesian Skyline coalescent prior [22] with a temporal bin of 5. Convergence and performance of MCMC chains were monitored with Tracer v1.7.2, ensuring effective sample sizes (ESSs) exceeded 200 for all parameters. MCMC simulations were executed as six independent runs of 50 million generations each (totaling 300 million generations), sampled every 2000 generations (25,000 states per chain), discarding the initial 20% as burn-in prior to combining logs and trees using LogCombiner. Parameter uncertainty was summarized by 95% of the highest posterior density (HPD) intervals. Maximum clade credibility (MCC) trees were generated using Tree Annotator v2.7.7 and visualized with FigTree v1.4.4.

### 2.5. Positive Selection Analysis

To identify selective pressures on RSV genomes, nonsynonymous (dN) and synonymous (dS) substitution rates per site were calculated using the Nei–Gojobori method with Jukes–Cantor correction, as implemented in MEGA v12 [23]. Genes displaying an overall dN/dS ratio indicative of positive selection (dN/dS ratio > 1) underwent further site-specific analysis using the Datamonkey web server (http://www.datamonkey.org/, accessed on 15 July 2025) [24]. This gene-level threshold was applied as a conservative prioritization step to focus codon-based testing. Four complementary methods—Single-Likelihood Ancestor Counting (SLAC), Fixed Effects Likelihood (FEL), Mixed Effects Model of Evolution (MEME), and Fast, Unconstrained Bayesian AppRoximation (FUBAR)—were employed to detect codon-specific positive selection [25,26,27]. Codon sites meeting significance thresholds (SLAC, FEL, MEME: *p* < 0.05; FUBAR: posterior probability > 0.95) were considered positively selected.

### 2.6. Average Shannon Entropy Calculation

To assess overall evolutionary patterns, we quantified genome-wide nucleotide and amino acid diversity by calculating site-specific average Shannon entropy values using the Entropy-One Tool (https://www.hiv.lanl.gov/content/sequence/ENTROPY/entropy_one.html, access on 18 July 2025). To evaluate subgroup differences, we compared site-level entropy distributions between RSV-A and RSV-B for each gene using Mann–Whitney U tests. To account for multiple comparisons across genes, *p*-values were adjusted with the Benjamini–Hochberg procedure, and results are reported as *q*-values. Genes with *q* < 0.05 were considered statistically significant. We also calculated 95% confidence intervals for mean entropy (Supplementary Table S4).

## 3. Results

### 3.1. Patient Demographics and Sample Distribution

The sample distribution was not uniform throughout 2018 to 2024, and RSV samples were not available in 2021 due to low prevalence. Across the study period, all available RSV-positive samples with accompanying metadata were collected, resulting in 1173 unique patient samples with an approximately 1:1 male-to-female ratio (Table 1). Most samples were from infants (333/1173, 28.4%), children 1–5 years (412/1173, 35.1%), and older adults ≥ 60 years (186/1173, 15.9%). A total of 576 (49.1%) patients had one or more comorbidities. The most prevalent comorbidities were cancer (465/1173, 39.6%), non-asthmatic chronic lung disease (377/1173, 32.1%), and immunosuppression (309/1173, 26.3%). Healthcare utilization varied by year, with emergency department visits and ICU-level care being consistently high. Hospital admissions and supplemental oxygen use declined noticeably after 2022. However, we could not directly quantify the contribution of changing testing frequency to the observed temporal trends in clinical severity.

**Figure 1:**
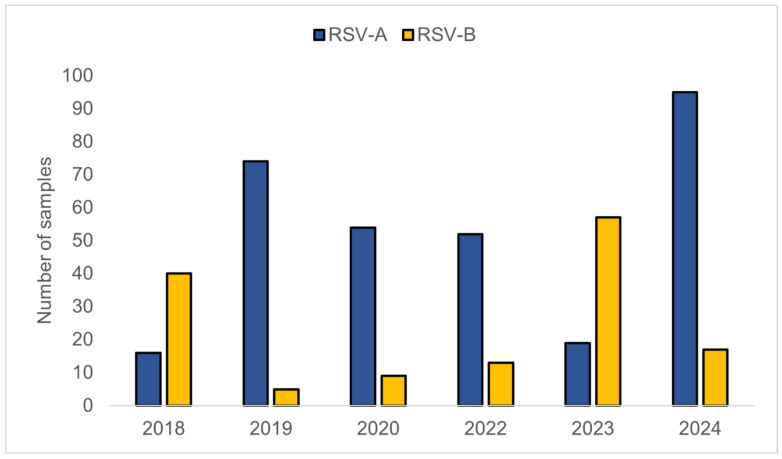
Temporal distribution of RSV-A (blue) and RSV-B (yellow) in samples collected between 2018 and 2024. No samples were available for 2021 due to low RSV prevalence.

For each respiratory season, we randomly sampled without replacement around 95 RSV-positive samples from the pool of eligible samples for sequencing. This number was chosen because it corresponds to exactly one sequencing run and, in some years, the total number of specimens archived was limited. Another consideration was to avoid overrepresenting any single year, which could artificially skew estimates of yearly genetic diversity in downstream analyses. Throughout the study timeframe, a total of 451 RSV genomes out of 570 genomes were successfully recovered, comprising 310 RSV-A (68.7%) and 141 RSV-B (31.3%) cases (Figure 1). Between 2018 and 2024, RSV-A and RSV-B exhibited fluctuating predominance. RSV-B dominated in 2018 (40 vs. 16 RSV-A) and 2023 (57 vs. 19 RSV-A), whereas RSV-A was the major subgroup in 2019 (74 vs. 5 RSV-B), 2020 (54 vs. 9 RSV-B), 2022 (52 vs. 13 RSV-B), and 2024 (95 vs. 17 RSV-B). The largest seasonal cohort was in 2024 (n = 112), whereas 2018 had the fewest samples (n = 56), largely due to inadequate RNA quality and low viral loads that limited sequencing success. Our specimens were derived from routine clinical testing within JHHS, and samples selected for sequencing as well as recovering high-quality genomes reflect changes in the JHHS respiratory testing volumes and RSV positivity trends [16,28,29].

### 3.2. Phylogenetic Analysis of RSV

**Figure 2:**
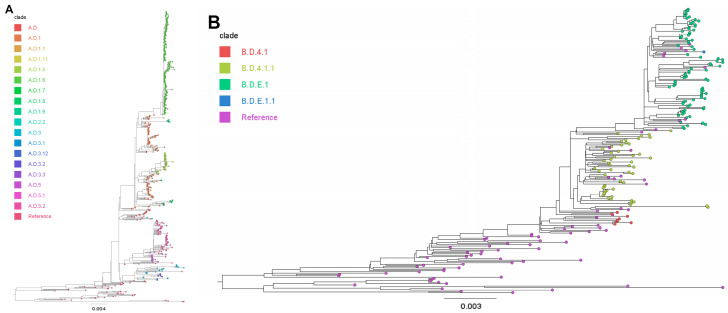
Rooted maximum likelihood phylogenetic trees of (**A**) RSV-A and (**B**) RSV-B based on whole-genome sequence alignments. Colored dots represent distinct clade, and light purple dots correspond to reference genomes. The branch-length scale differs between panels (**A**,**B**).

Phylogenetic analysis revealed considerable genetic diversity in RSV, identifying a total of 15 RSV-A clades and 5 RSV-B clades circulating in Maryland from 2018 to 2024 (Supplementary Table S5). RSV clade circulation patterns demonstrated notable yearly fluctuations (Table 2). RSV-B predominated in 2018 (40/56, 71.4%), primarily driven by clade B.D.4.1.1 (32/40, 80.0%), while RSV-A cases were less frequent but exhibited greater clade diversity, led by A.D (10/16, 62.5%). RSV-A became predominant in subsequent years, particularly in 2019 (74/79, 93.6%) and 2020 (54/63, 85.7%), driven largely by the clade A.D.1 (61/74, 82.4% in 2019; 43/54, 79.6% in 2020), whereas RSV-B detections drastically declined and consisted solely of B.D.4.1.1. By 2022, RSV-A continued as the primary subgroup (52/65, 80.0%) but experienced clade diversification, with A.D.5.2 emerging as the predominant clade (24/52, 46.2%), and RSV-B was dominated by B.D.E.1 (12/13, 92.3%). RSV-B further expanded to dominance in 2023 (57/76, 75.0%), largely comprising clade B.D.E.1 (53/57, 93.0%), while RSV-A decreased in prevalence, primarily represented by A.D.5.2 (7/19, 36.8%). In 2024, RSV-A again became highly dominant (95/112, 84.8%), driven notably by clade A.D.1.6 (62/95, 65.3%), whereas RSV-B circulation was limited and predominantly included clade B.D.E.1 (16/17, 94.1%). All RSV-A sequences obtained during the study period were classified as the ON1 genotype (also referred to as GA2.3.5 in the G-gene clade nomenclature; Figure 2A), whereas all RSV-B sequences were assigned to the BA9 genotype (also referred to as GB5.0.5a; Figure 2B).

### 3.3. Shannon Entropy, Estimated Evolutionary Rate, and Bayesian Dated Phylogenetic Tree

**Figure 3:**
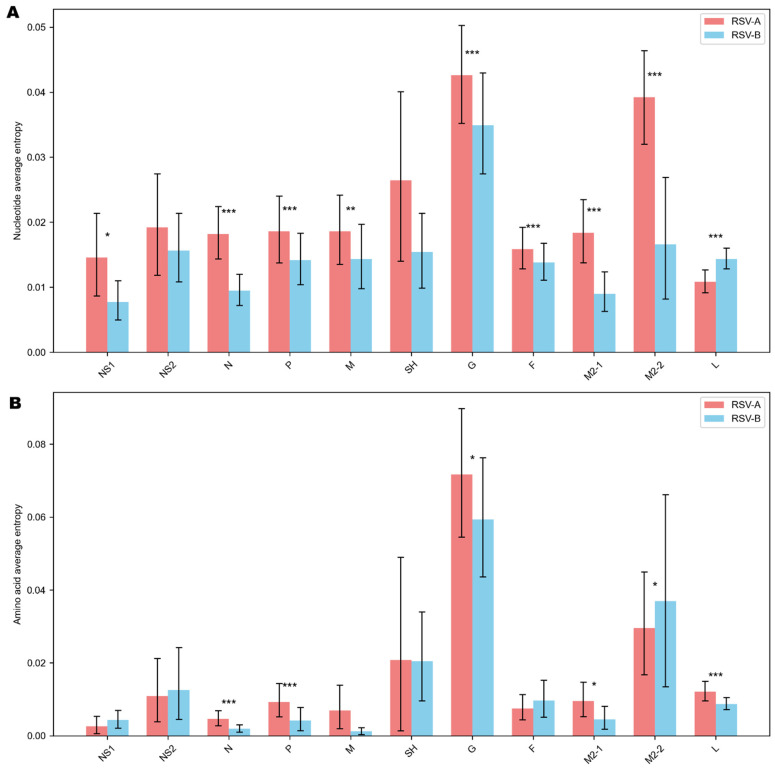
Nucleotide (**A**) and amino acid (**B**) entropy of RSV-A and RSV-B. Bar plots show mean nucleotide and amino acid entropy values for each RSV gene, with error bars representing 95% confidence intervals. Red bars correspond to RSV-A, and blue bars correspond to RSV-B. Asterisks denote statistically significant differences between subgroups (*q* < 0.05 = *; *q* < 0.01 = **; *q* < 0.001 = ***).

At the nucleotide level (Figure 3A), the G gene exhibited the highest average entropy for both subgroups (0.045 in RSV-A; 0.035 in RSV-B). The SH (0.026) and M2-2 (0.039) genes of RSV-A also showed elevated average nucleotide entropy relative to other genes such as L (0.010). At the amino acid level (Figure 3B), the G protein again displayed the greatest variability, with an average entropy of 0.078 in RSV-A and 0.060 in RSV-B. Beyond G, only the M2-2 protein (0.037) demonstrated moderate amino acid diversity in RSV-B.

Between subgroup comparisons of site-level entropy distributions within each gene showed that RSV-A had significantly greater nucleotide diversity than RSV-B in F, G, M, M2-1, M2-2, N, NS1, and P (q < 0.05), whereas RSV-B showed higher nucleotide entropy than RSV-A in L (q < 0.05). At the amino acid level, RSV-A was significantly more diverse than RSV-B in G, L, M2-1, N, and P, while RSV-B showed greater variability than RSV-A in M2-2 (q < 0.05). Although SH and F showed slightly higher average entropies in RSV-B, these differences were not statistically significant.

**Figure 4:**
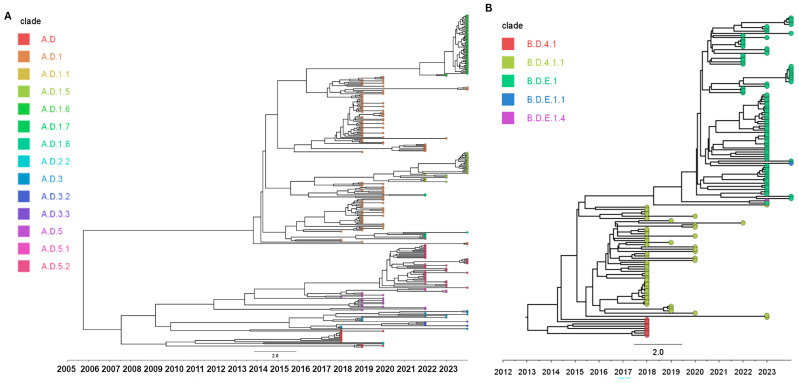
Time-scaled whole-genome phylogenies of (**A**) RSV-A and (**B**) RSV-B inferred using a Bayesian MCMC framework. The x-axis shows calendar year, and branch lengths reflect inferred time. Colored dots indicate clade assignments as listed in the legends.

The mean evolutionary rate of the RSV-A dataset, estimated using an uncorrelated lognormal relaxed molecular clock, was 7.07 × 10^−4^ substitutions per site per year (95% HPD: 5.52 × 10^−4^ to 8.56 × 10^−4^). The estimated time to the most recent common ancestor (tMRCA) for all our RSV-A sequences was approximately 18.5 years before the most recent sample (95% HPD: 12.8–25.5 years) (Figure 4A). For all our RSV-B sequences, the mean evolutionary rate was slightly higher compared to our RSV-A samples, at 1.02 × 10^−3^ substitutions/site/year (95% HPD: 8.01 × 10^−4^–1.06 × 10^−3^), with a tMRCA of approximately 10.9 years (95% HPD: 9.78–12.16 years) (Figure 4B). Since all RSV-A genomes recovered in this study belonged to the ON1-derived genotype and all RSV-B genomes belonged to BA9, these tMRCA estimates reflect the most recent common ancestors of the sampled ON1 and BA9 viruses in our dataset, rather than the original emergence times of ON1 or BA9 globally.

### 3.4. Population Dynamic Analysis

**Figure 5:**
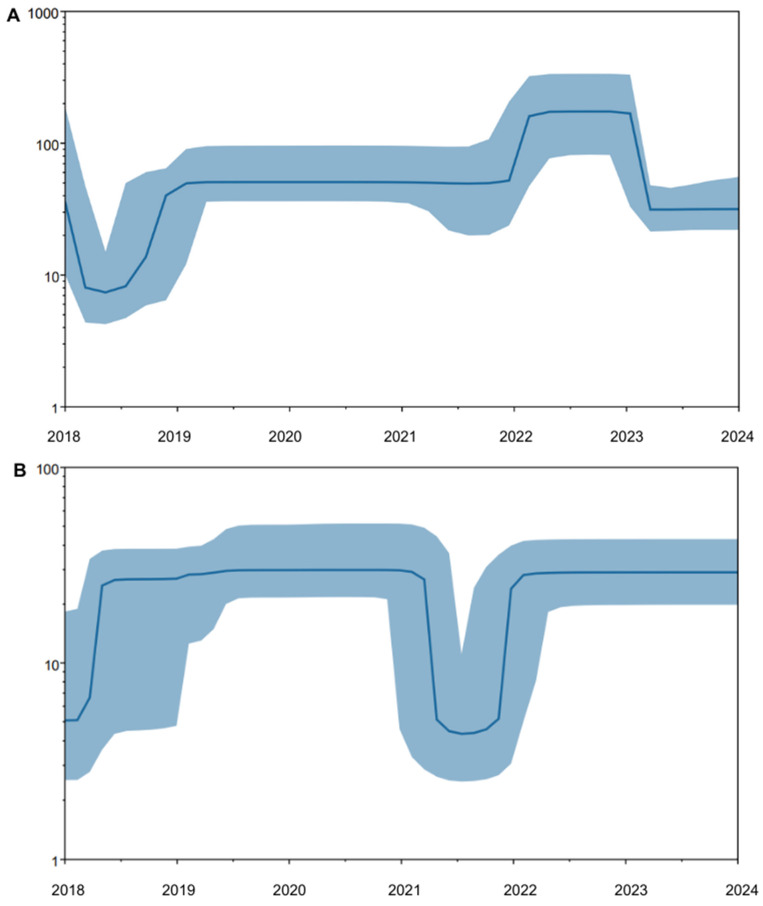
Bayesian Skyline Plots to illustrate temporal changes in the effective population size of (**A**) RSV-A and (**B**) RSV-B from 2018 to 2024. The median effective population size is shown by the solid blue line, while the surrounding shaded area represents the 95% HPD interval.

To explore changes in RSV genetic diversity over time, we performed BSP reconstruction using a coalescent-based model implemented in BEAST2. The analysis revealed substantial temporal fluctuations in the effective population size (Ne) between 2018 and 2024 (Figure 5A). Ne was broadly stable across the study period with a single pronounced expansion peak in each subgroup, and we interpret these results as changes in genetic diversity. A sharp decline in genetic diversity of RSV-A was observed between 2018 and 2019, followed by a period of relative stability from 2019 to 2021. A notable expansion of RSV-A occurred in early 2022, peaking throughout the year. This was followed by a sharp decline in RSV-A diversity in 2023 and stabilization at a lower level in 2024. BSP analysis also revealed substantial temporal variation in the effective population size of RSV-B between 2018 and 2024 (Figure 5B). A marked expansion in genetic diversity of RSV-B was observed in mid-2018 to -2019, followed by a sustained period of a relatively stable population size through 2021. However, a dramatic reduction in RSV-B in effective population size occurred during the 2021–2022 period. This population bottleneck reached its lowest point in late 2021, after which the RSV-B population rebounded rapidly, returning to pre-pandemic diversity levels by 2022 and remaining stable in 2023 and 2024. Using a Bayesian Skyline model with 5 temporal bins, the inferred Ne trajectories were broadly stable over time with a single prominent expansion peak for each subgroup. We interpret these skyline constructions as coarse changes in genetic diversity rather than fine-scale temporal fluctuations. Because no RSV genomes were available from 2021, skyline estimate through 2021 is only informed by sequences sampled before and after the gap and therefore may have reduced temporal resolution within this interval.

### 3.5. Site-Specific Selection Analysis and Positive Selection Sites

To investigate selective pressures on the RSV genome, the average ratio of dN/dS was calculated for each gene (Table 3). Multiple genes exhibited average dN/dS ratios > 1, suggesting possible diversifying selection. In RSV-A, the M (1.016), SH (1.218), G (1.149), and F (1.066) genes showed signs of positive selection. Similarly, in RSV-B, the SH (1.116), G (1.113), F (1.151), and M2-2 (1.406) genes demonstrated evidence of positive selection. Other genes showed either negative or neutral selection.

Site-specific analyses further supported these findings. For the G protein, codons 71, 133, 154, 178, 225, 255, 273, and 314 in RSV-A, and 217, 276, and 285 in RSV-B were detected using at least one method (SLAC, FEL, FUBAR, or MEME). In RSV-A, only codon 273 in the G protein was supported by all four models, providing strong evidence of diversifying selection (Table 4). In RSV-B, codon 217 in the G gene was the only site identified as positively selected by all models. Additionally, only codon 245 in the RSV-A F gene was detected by MEME, suggesting episodic selection. No positively selected codons were identified in other proteins, including M, SH, or M2-2, by site-level analysis despite high average dN/dS ratios in some of these genes.

## 4. Discussion

The estimated evolutionary rate for RSV-A in our study falls within the expected range and is consistent with previously reported values, which generally fall between 6.72 × 10^−4^ and 7.69 × 10^−4^ substitutions/site/year based on a multi-country whole-genome phylodynamic analysis [30]. Despite higher average entropy at the nucleotide level and the amino acid level in RSV-A, the mean evolutionary rate for RSV-A in our study was slightly lower than that of RSV-B, suggesting that RSV-B may be evolving more rapidly within the local population. Consistent with prior studies from China, Italy, Japan, and South Korea, RSV-B displayed higher evolutionary rates than RSV-A, with reported values ranging from ~1.0 × 10^−3^ to 4.6 × 10^−3^ substitutions/site/year [31,32,33,34]. Previous Kenyan and Korean studies have also reported that RSV-B BA9 exhibited higher evolutionary rates than RSV-A ON1 locally [35,36]. However, a study from Senegal reported a higher ON1 substitution rate when compared to the BA9 genotype [37]. These differences likely reflect the use of gene-specific datasets, which often yield elevated rate estimates compared to whole-genome analyses such as ours, as well as potential regional variation in epidemic dynamics and the specific time periods analyzed. This could also be due to RSV-A exhibiting a broader quasispecies cloud and a larger variant pool; however, many of those variants may remain at a low frequency and fail to achieve selective advantage [38].

Between 2018 and 2024, RSV circulation was globally dominated by ON1-derived RSV-A and BA9-derived RSV-B, with geography-specific fluctuations among their clades but no evidence of replacement by novel genotypes [39]. Since 2020, the adoption of a finer clade classification has enabled studies to resolve clade dynamics with greater resolution [4]. Our analysis revealed patterns largely consistent with international reports but also exhibited distinct local features. RSV-B in our region was stably dominated by clade B.D.E.1 from 2022 through 2024, paralleling its rise in Minnesota, Beijing, Sicily, and Mexico, and consistent with global surveillance analyses identifying B.D.E.1 as a major clade driving RSV-B spread worldwide [39,40,41,42,43,44]. For RSV-A, clade A.D.5.2 was common during 2022–2023, in line with observations from these same regions, but by 2024 it was largely dominated locally by A.D.1.6, a clade not yet reported as dominant elsewhere. The subgroup fluctuations and clade turnover here likely reflect post-COVID changes in transmission. Non-pharmaceutical interventions can suppress circulation and reduce diversity, amplifying founder effects and re-seeding dynamics when mixing resumes, producing short-term dominance of particular clades even without clear evidence of a major adaptive advantage. We therefore interpret the observed subgroup predominance and clade turnover as results of changing population susceptibility and contact patterns and stochastic re-seeding after bottlenecks, rather than as evidence for a selectively drive genotype replacement.

The older tMRCA estimated for RSV-A compared to RSV-B in our dataset likely reflects distinct evolutionary histories of their dominant circulating clades, ON1 and BA9, respectively. ON1, first detected in 2010, is thought to have emerged several years earlier and may have circulated cryptically before global dissemination, as exemplified in emerging pathogens such as SARS-CoV-2 [45]. Its prolonged persistence and the presence of multiple co-circulating clades likely contributed to the deeper ancestral structure observed in RSV-A. In contrast, the more recent tMRCA of RSV-B may be attributed to stronger population bottlenecks and rapid clade turnover within the BA genotype [46]. The dominance of BA9 in recent years may reflect a clade replacement event that erased older genetic diversity, resulting in a more recent common ancestor. Nonetheless, our tMRCA estimates for RSV-A and RSV-B align with other regional studies. Kenyan whole-genome analysis placed the ON1 ancestor around 2005, while U.S. genomic data from Massachusetts traced RSV-A to 2009 and RSV-B to 2016, and Washington State surveillance similarly suggested that both subgroups had been diversifying for over a decade [47,48,49].

The population dynamics we observed in Maryland closely mirror global reports of RSV disruptions and rebounds during the COVID-19 pandemic. In Australia, RSV circulation was nearly absent during the winter of 2020, followed by large out-of-season outbreaks in 2021–2022, with genomic analyses showing reduced clade diversity and the expansion of a few RSV-A clades [50]. Similar patterns were described in New Zealand, where RSV was nearly eliminated in 2020–2021 and then reintroduced after border reopening, initially with low diversity that broadened as circulation resumed [51]. In the United States, national surveillance and regional genomic studies documented off-season resurgences in 2021 and intense early epidemics in 2022, driven by multiple co-circulating clades rather than emergence of a novel strain [49,52,53]. Our findings of RSV-A diversity in 2022 and RSV-B predominance in 2023 align with these national patterns. Across Europe, delayed and shifted epidemics in 2020–2021 were followed by RSV-B predominance in 2022–2023, as observed in France, Iceland, and Italy, paralleling the subgroup shifts we detected in Maryland [54,55,56]. However, the number of genomes sequenced varied substantially by year, skyline-derived Ne estimates for sampled years are less precise and may be over-smoothed.

Although preliminary average dN/dS analyses suggested possible positive selection sites in the G, F, SH, M, and M2-2 genes, only the G and F genes consistently demonstrated robust evidence of positive selection across four detection models. Given their roles as major antigenic targets, it is not surprising that the G and F genes are under diversifying selection [57]. In contrast, internal proteins such as N and P are functionally constrained and thus evolve under purifying selection to preserve essential viral replication and assembly functions [58].

In this study, fewer positively selected sites in the G gene were identified compared to previous reports, potentially due to stringent criteria applied in our analysis (*p*-value < 0.05, posterior probability > 0.95) [44,59]. Despite this quantitative difference, the positively selected sites identified consistently mapped to the first and second hypervariable regions of the G gene as reported by other RSV phylodynamic studies [40,59]. Eight G codons were found to be positively selected for RSV-A, with codon 273 being the only site identified as significant by all four models. This codon has been previously recognized in multiple studies as a hotspot for mutations [60,61,62]. Notably, codon 273 corresponds to an N-glycosylation site that is completely lost in the ON1 genotype [63,64]. Glycosylation can be under significant selective pressure from factors such as immune evasion, so the loss of glycosylation at this site may expose neighboring residues to immune surveillance [65]. Glycosylation of the RSV G protein plays a key role in shielding antigenic epitopes from neutralizing antibodies; its removal likely reflects a trade-off between immune evasion and structural flexibility [66]. However, no mutational analysis was performed to validate the impact of substitutions at this position. Four of the positively selected sites (codon 133, 225, 255, and 273) in RSV-A strains were located within previously predicted ON1 B-cell epitope regions, suggesting their potential immunological relevance [67]. Some positively selected G sites (codon 71, 225, 273, 314) were consistent with previous studies, with some sites (codon 133, 154, 178, 255) being first detected [36,59,68,69]. However, some studies suggest that the high entropy and genetic diversity of the RSV G protein may not be primarily driven by immune-mediated antigenic drift but instead may arise from neutral evolutionary processes such as genetic drift following population bottlenecks [70]. One contributing factor of predicted positive selection sites is the limited immunogenicity of strain-specific epitopes in the G protein’s C-terminal region, which tend to elicit weak neutralizing antibody responses [8]. Therefore, most potent and broadly neutralizing responses target the conserved prefusion F protein, as well as the central conserved domain of G [71,72]. Among RSV-B strains, three G sites were under positive selection, with codon 217 identified as significant across all four models. Codon 217 exhibited high frequency and Shannon entropy in a study from Cameroon, further supporting its role as a variable immunogenic region [73]. Codons 217, 276, and 285 in the RSV-B G protein have been repeatedly identified under positive selection [62,69,74]. However, we did not perform experimental functional validation such as site-directed mutagenesis to directly test the impact of substitutions at these positions of interest.

In our analysis of the RSV-A F gene, only codon 245 showed evidence of episodic selection by the MEME method. Although this site was not identified by all methods, it remains noteworthy to highlight because the F protein is relatively conserved and represents a key antigenic target for vaccines and monoclonal antibodies. Codon 245 lies within the F1 subunit of the F protein but outside the six major antigenic sites (Ø, I, II, IV, V, and VI) [57]. It does not overlap with any well-characterized neutralizing epitopes [75]. This suggests that adaptive change at this site may occur in more peripheral regions of F without directly altering mapped antibody-binding sites. In line with this, recent studies of the F protein have shown that certain residues outside canonical epitopes can act as conformational switches, altering the local or global dynamics of F and thereby influencing how neutralization epitopes are displayed to the immune system [7,76]. The identification of codon 245 as a positively selected site is therefore intriguing, as it has not been highlighted in previous reports. This novel site could reflect a localized immune pressure or functional adaptation unique to the viruses in our dataset. No experimental mutagenesis was performed on codon 245, so the impact of its variation remains unverified.

This study has several limitations. Uneven sampling across years may have influenced our phylodynamic and selection inferences by over-weighting clades from more densely sampled periods and under-representing circulating diversity in sparsely sampled years. The absence of 2021 genomes create a key temporal gap during the COVID-19 transition, limiting our ability to distinguish persistence from re-introduction and potentially smoothing skyline-derived Ne trajectories across 2020–2022. Limited subgroup representation from fewer RSV-B genomes reduces precision for subgroup-specific evolutionary rate and tMRCA estimates and lowers power to detect episodic or low-frequency positively selected sites. Since vaccination status and receipt of monoclonal antibody prophylaxis were unavailable and the dataset reflects medically attended infections, we could not stratify analyses by immune exposure or perform formal clinical-genomic association testing, which limits interpretation of antigenic selection signals and clade-severity relationships. Finally, codon-based selection methods can be sensitive to alignment uncertainty and uneven temporal sampling; thus, identified sites should be viewed as hypothesis-generating without experimental validation.

Despite these limitations, our analysis captures the evolutionary trajectories of RSV-A and RSV-B circulating in Maryland over seven years, demonstrating subgroup shifts, clade replacement, and variable selective pressures. Our findings also have direct implications for RSV preparedness and prevention policy. Since subgroup fluctuation and clade turnover in Maryland occurred largely within established ON1- and BA9-derived clades rather than through replacement by a novel genotype, the greatest standardized genomic surveillance is temporally even and linked to routine diagnostics, rather than episodic sequencing only during peaks. Integrating sequencing outputs with immunization and prophylaxis data would strengthen prevention strategies because locally informed surveillance can help optimize the timing and targeting with clinical guidance.

## Figures and Tables

**Table 1:** Patient cohort and characteristics from 2018 to 2024.

Characteristics	Number of Patients (% Cohort)					
	2018	2019	2020	2022	2023	2024
Unique patients	277 (23.6)	461 (39.3)	149 (12.7)	79 (6.7)	95 (8.1)	112 (9.5)
Female	123 (44.4)	224 (48.6)	79 (53.0)	46 (58.2)	46 (48.4)	58 (51.8)
Male	154 (55.6)	237 (51.4)	72 (47.0)	33 (41.8)	49 (51.6)	54 (48.2)
Age						
0–11 months	59 (21.3)	138 (29.9)	54 (36.2)	8 (10.1)	17 (17.9)	36 (32.1)
1–5	70 (25.3)	175 (38.0)	39 (26.2)	32 (40.5)	34 (35.8)	56 (50.0)
6–17	18 (6.5)	27 (5.9)	5 (3.4)	11 (13.9)	19 (20.0)	8 (7.1)
18–59	64 (23.1)	77 (16.7)	27 (18.1)	16 (20.3)	14 (14.7)	9 (8.0)
>=60	66 (23.8)	44 (9.5)	24 (16.1)	12 (15.2)	11 (11.6)	3 (2.7)
Comorbidities						
Asthma	16 (5.8)	29 (6.3)	3 (2.0)	0	13 (13.7)	7 (6.3)
Atrial fibrillation	30 (10.8)	23 (5.0)	14 (9.4)	2 (2.5)	3 (3.2)	0
Cancer	116 (41.9)	124 (26.9)	50 (33.6)	24 (30.4)	27 (28.4)	18 (16.1)
Cerebrovascular disease	34 (12.3)	31 (6.7)	18 (12.1)	3 (3.8)	9 (9.5)	3 (2.7)
Coronary artery disease	76 (27.4)	59 (12.8)	26 (17.4)	8 (10.1)	14 (14.7)	6 (5.4)
Diabetes	57 (20.6)	48 (10.4)	24 (16.1)	10 (12.7)	12 (12.6)	5 (4.5)
Heart failure	61 (22.0)	45 (9.8)	18 (12.1)	5 (6.3)	8 (8.4)	4 (3.6)
Hypertension	106 (38.3)	100 (21.7)	38 (25.5)	18 (22.8)	17 (17.9)	8 (7.1)
Immunosuppression	121 (43.7)	120 (26.0)	55 (36.9)	7 (8.9)	25 (26.3)	16 (14.3)
Kidney disease	88 (31.8)	76 (16.5)	28 (18.8)	8 (10.1)	14 (14.7)	5 (4.5)
Non-asthmatic lung disease	131 (47.3)	167 (36.2)	45 (30.2)	18 (22.8)	35 (36.8)	24 (21.4)
Smoker	37 (13.4)	24 (5.2)	16 (10.7)	3 (3.8)	7 (7.4)	3 (2.7)
Pregnant	1 (0.4)	10 (2.2)	5 (3.4)	1 (1.3)	0	0
Emergency department visit	187 (67.5)	357 (77.4)	105 (70.5)	7 (8.9)	84 (88.4)	107 (95.5)
Admitted	145 (52.3)	195 (42.3)	73 (49.0)	3 (3.8)	32 (33.7)	27 (24.1)
0–11 months	34 (57.6)	69 (50.0)	29 (53.7)	1 (12.5)	5 (29.4)	9 (25.0)
1–5	20 (28.6)	56 (32.0)	10 (25.6)	1 (3.1)	6 (17.6)	7 (12.5)
6–17	9 (50.0)	8 (29.6)	2 (40.0)	0	5 (26.3)	5 (62.5)
18–59	37 (57.8)	37 (48.1)	14 (51.9)	0	7 (50.0)	4 (44.4)
>=60	45 (68.2)	25 (56.8)	18 (66.7)	1 (8.3)	9 (81.8)	2 (66.7)
ICU-level care	43 (15.5)	60 (13.0)	22 (14.8)	0	5 (5.3)	6 (5.4)
Supplemental Oxygen	110 (39.7)	154 (33.4)	49 (32.9)	4 (5.1)	24 (25.3)	18 (16.1)

**Table 2:** Dominant RSV Clades between 2018 and 2024.

Year	Subgroup	Total	Dominant Clade	Number of Samples (% Cohort)
2018	A	16	A.D	10 (62.5)
	B	40	B.D.4.1.1	32 (80.0)
2019	A	74	A.D.1	61 (82.4)
	B	5	B.D.4.1.1	5 (100.0)
2020	A	54	A.D.1	43 (79.6)
	B	9	B.D.4.1.1	9 (100.0)
2022	A	52	A.D.5.2	24 (46.2)
	B	13	B.D.E.1	12 (92.3)
2023	A	19	A.D.5.2	7 (36.8)
	B	57	B.D.E.1	53 (93.0)
2024	A	95	A.D.1.6	62 (65.3)
	B	17	B.D.E.1	16 (94.1)

**Table 3:** Mean dN/dS ratio for each gene in RSV-A and RSV-B.

Gene	Subgroup	Length (bp)	Average dN/dS Ratio	Selection
*NS1*	A	420	0.046	Negative
	B	420	0.056	Negative
*NS2*	A	375	0.061	Negative
	B	375	0.293	Negative
*N*	A	1176	0.017	Negative
	B	1176	0.013	Negative
*P*	A	726	0.022	Negative
	B	726	0.042	Negative
*M*	A	771	1.016	Positive
	B	771	1.000	Neutral
*SH*	A	195	1.218	Positive
	B	198	1.116	Positive
*G*	A	969	1.149	Positive
	B	984	1.113	Positive
*F*	A	1725	1.066	Positive
	B	1725	1.151	Positive
*M2-1*	A	585	0.049	Negative
	B	588	0.449	Negative
*M2-2*	A	273	0.952	Negative
	B	273	1.406	Positive
*L*	A	6498	0.140	Negative
	B	6501	0.048	Negative

**Table 4:** G- and F-protein positive selection.

Protein	Subgroup	Site	SLAC	FEL	FUBAR	MEME	Site of Reference
G	RSV-A	71	Y	N	N	N	71
		133	N	N	N	Y	133
		154	N	N	N	Y	154
		178	N	N	N	Y	178
		225	Y	N	N	N	225
		255	N	Y	N	Y	255
		273	Y	Y	Y	Y	273
		314	Y	N	N	N	314
	RSV-B	217	Y	Y	Y	Y	217
		276	N	N	N	Y	276
		285	N	Y	Y	Y	285
F	RSV-A	245	N	N	N	Y	245

## Data Availability

All studied RSV genomes have been deposited in the GISAID database and are available in Supplementary Table S6.

## Supplementary Materials

The following supporting information can be downloaded at: https://www.mdpi.com/article/10.3390/v18030331/s1, Table S1: Primers and probe for RT-qPCR and RT-PCR; Table S2: Reference genomes from GenBank used for the phylogenetic analysis; Table S3: Clock model comparison with marginal likelihood and ESS on the whole-genome of RSV-A and RSV-B sequences; Table S4: Gene-specific nucleotide and amino acid entropy in RSV-A and RSV-B; Table S5: Distribution of RSV Subtypes and Clades from 2018 to 2024 Cohort; Table S6: GISAID Accession Numbers for RSV-A and RSV-B Sequences.
