# Supplementary material for: Whole-Genome Phylodynamic Analysis of Respiratory Syncytial Virus—Maryland, USA, 2018–2024
Source: Viruses. 2026 Mar 7;18(3):331. doi: 10.3390/v18030331 (PMC13030589; doi:10.3390/v18030331)
Supplement: Supplementary file 1 [file viruses-18-00331-s001.zip › Supplementary Table S1.pdf]

Supplementary Table S1. Primers and probe for RT-qPCR and RT-PCR

RT-qPCR Primers and Probe		
**Primer**	**Sequence (5’-3’)**	**Final concentration (nM)**
Forward	GGCAAATATGGAAACATACGTGAA	400
Reverse	TCTTTTTCTAGGACATTGTAYTGAACAG	400
Probe	FAM-CTGTGTATGTGGAGCCTTCGTGAAGCT-Iowa Black FQ	200
**RT-PCR Primers**		
**Pool 1**		
**Amplicon**	**Primer sequence (5'–3')**	**Final concentration (nM)**
Amplicon 1 Forward	AAAAATGCGTACWACAAACTTGC	400
Amplicon 1 Reverse	TTGATTGMAAAWCGTGTAGCT	400
Amplicon 3 Forward	TGATGCATCAATATCTCAAGTC	400
Amplicon 3A Reverse	AGGACTTTCTTTATACTAGCTG	200
Amplicon 3B Reverse	AGGACTTTTTTGATACTGGCTG	200
**Pool 2**		
**Amplicon**	**Primer sequence (5'–3')**	**Final concentration (nM)**
Amplicon 2 Forward	GCCACARAGTCAATTYATAGTAG	200
Amplicon 2 Reverse	TGTRACTGGTGTGYTTYTGG	200
Amplicon 4 Forward	CTCAAGCAGATTATTTGYTAGCA	400
Amplicon 4A Reverse	TTGAATACAATGTTAGTGTGTAGC	400
Amplicon 4B Reverse	GTTGTAAATGCACATGTGTGATTG	400
