# Supplementary material for: Whole-Genome Phylodynamic Analysis of Respiratory Syncytial Virus—Maryland, USA, 2018–2024
Source: Viruses. 2026 Mar 7;18(3):331. doi: 10.3390/v18030331 (PMC13030589; doi:10.3390/v18030331)
Supplement: Supplementary file 1 [file viruses-18-00331-s001.zip › Supplementary Table S2.pdf]

Supplementary Table S2. Reference genomes from GenBank used for the phylogenetic analysis

**RSV-A Reference**
hRSV/A/South_Africa/NICD-R00863/2023|EPI_ISL_17559364|2023-01-30
hRSV/A/Senegal/0004/2022|EPI_ISL_18228290|2022-01-04
hRSV/A/Italy/LOM-34/2022|EPI_ISL_18446762|2022-01-07
hRSV/A/Spain/CT-HUVH-991848/2023|EPI_ISL_18682169|2023-01-05
hRSV/A/Spain/CT-HUVH-281345/2022|EPI_ISL_18682390|2022-01-04
hRSV/A/Kuwait/KUNIV-52536/2022|EPI_ISL_18851841|2022-01-03
hRSV/A/USA/MO-USAFSAM-R00056/2023|EPI_ISL_19069901|2023-01-05
hRSV/A/Canada/ON-KHS-00878-v4/2024|EPI_ISL_19320833|2024-01-01
hRSV/A/France/un-HMN-014-0006/2024|EPI_ISL_19630772|2024-01-01
hRSV/A/China/JN-CDC-10/2024|EPI_ISL_20264416|2024-01-01
hRSV/A/South_Africa/NICD-R00749/2021|EPI_ISL_11055716|2021-01-19
hRSV/A/Australia/QLD-RBWH022/2021|EPI_ISL_11817025|2021-01-18
hRSV/A/England/200260431/2020|EPI_ISL_1520412|2020-01-07
hRSV/A/Spain/un-DP-SE02-0007-D02/2019|EPI_ISL_15753315|2019-01-18
hRSV/A/Canada/un-UMC-25676/2020|EPI_ISL_18061092|2020-01-07
hRSV/A/Netherlands/un-UMC-26139/2020|EPI_ISL_18061555|2020-01-07
hRSV/A/Spain/un-UMC-26321/2020|EPI_ISL_18061737|2020-01-07
hRSV/A/Australia/VIC-RCH150/2018|EPI_ISL_1834133|2018-07-28
hRSV/A/France/ARA-HCL021010287401/2021|EPI_ISL_18789149|2021-01-18
hRSV/A/Brazil/un-UMC-550101050/2018|EPI_ISL_19048146|2018-07-30
hRSV/A/France/un-UMC-330102012/2019|EPI_ISL_19049252|2019-01-18
hRSV/A/New_Zealand/IESR-CF0681/2018|EPI_ISL_19207149|2018-07-26
hRSV/A/Mongolia/NCCD009/2019|EPI_ISL_2543834|2019-01-18
hRSV/A/Canada/un-CAN2234/1995|EPI_ISL_15820266|1995
hRSV/A/Japan/KU-001/1980|EPI_ISL_17782655|1980
hRSV/A/Australia/un-WIMR-VR26K/1956|EPI_ISL_18090682|1956-01-01
hRSV/A/Japan/KU-0001/1990|EPI_ISL_18334078|1990
hRSV/A/Japan/CiRA-28-30/1998|EPI_ISL_19696283|1998-07-15
hRSV/A/USA/92I-094A-01/1992|EPI_ISL_2543991|1992
hRSV/A/USA/79I-039A-01-1979/1979|EPI_ISL_2543994|1979
hRSV/A/USA/85E-170-01/1985|EPI_ISL_2543996|1985
hRSV/A/USA/96E-128-01/1996|EPI_ISL_2544002|1996
hRSV/A/USA/96I-029A-01-01/1996|EPI_ISL_2544007|1996
hRSV/A/USA/81E-078-01-1977/1977|EPI_ISL_2544010|1977
hRSV/A/USA/88I-141A-01-01/1987|EPI_ISL_2544012|1987
hRSV/A/USA/MC256/1988|EPI_ISL_2544014|1988
hRSV/A/Australia/A2/1961|EPI_ISL_2544032|1961
hRSV/A/Uruguay/Mon-1-98/1998|EPI_ISL_2579616|1998
hRSV/A/USA/MC246/1985|EPI_ISL_2582521|1985

hRSV/A/USA/MC214/1980|EPI_ISL_2582923|1980
hRSV/A/USA/MC263/1994|EPI_ISL_2582949|1994
hRSV/A/USA/MC211/1980|EPI_ISL_2582954|1980
hRSV/A/Argentina/BA-HNRG-301/2017|EPI_ISL_1074053|2017-02-22
hRSV/A/Morocco/20-15/2014|EPI_ISL_15120677|2014-11-17
hRSV/A/Morocco/296-16/2016|EPI_ISL_15120730|2016-01-21
hRSV/A/Kenya/59165/2007|EPI_ISL_15752014|2007
hRSV/A/Kenya/58560/2007|EPI_ISL_15752016|2007
hRSV/A/Brazil/un-38608/2010|EPI_ISL_15752029|2010
hRSV/A/Brazil/un-37858/2008|EPI_ISL_15752059|2008
hRSV/A/Mozambique/MAP-INS014/2017|EPI_ISL_1647462|2017-02-14
hRSV/A/Germany/BE-00-02128/2000|EPI_ISL_17995593|2000-02-11
hRSV/A/Germany/NI-01-02679/2001|EPI_ISL_17995595|2001-03-29
hRSV/A/Germany/RP-02-02688/2002|EPI_ISL_17995600|2002-04-25
hRSV/A/Germany/BE-04-01144/2004|EPI_ISL_17995606|2004-01-21
hRSV/A/Germany/BE-05-00962/2005|EPI_ISL_17995613|2005-01-11
hRSV/A/Spain/CT-HUVH-141621/2015|EPI_ISL_18682262|2015-01-02
hRSV/A/USA/IL-NU-026/2018|EPI_ISL_18939432|2018-01
hRSV/A/USA/TX-GLS-79254/2004|EPI_ISL_19477573|2004
hRSV/A/United_Kingdom/GLS-NU0409/2009|EPI_ISL_19556975|2009
hRSV/A/USA/PA-MGEL-00029/2018|EPI_ISL_19794721|2018-01
hRSV/A/China/ZJ-CDC-16S028/2016|EPI_ISL_19814925|2016-01-25
hRSV/A/Jordan/JOR-C2356/2012|EPI_ISL_2543936|2012-01-26
hRSV/A/Vietnam/VN-733-10-09/2009|EPI_ISL_2543943|2009-10-28
hRSV/A/Italy/LOM-ITA-120/2009|EPI_ISL_2543952|2009-03-19
hRSV/A/Netherlands/02-017863/2002|EPI_ISL_2543956|2002-06-29
hRSV/A/Argentina/170/2005|EPI_ISL_2544044|2005-06-21
hRSV/A/Argentina/177/2006|EPI_ISL_2544046|2006-06-06
hRSV/A/Peru/PER-FLA0535/2008|EPI_ISL_2544061|2008-02-29
hRSV/A/India/A-NIV1114046-11/2011|EPI_ISL_2579857|2011
hRSV/A/USA/A-US-BID-V8376/2003|EPI_ISL_2579892|2003
hRSV/A/Philippines/TTa-12-054/2012|EPI_ISL_2582181|2012-02-08
hRSV/A/Australia/A-NSW-WM0279A-14/2014|EPI_ISL_2582223|2014-09-11
hRSV/A/USA/A-US-BID-V8481/2001|EPI_ISL_2582457|2001
hRSV/A/USA/A-US-BID-V8484/2001|EPI_ISL_2582460|2001
hRSV/A/China/LZ01-09/2009|EPI_ISL_2582563|2009-09-01
hRSV/A/USA/A-US-BID-V7346/2002|EPI_ISL_2582729|2002
hRSV/A/USA/A-WI-629-9-06-07/2006|EPI_ISL_2583065|2006
hRSV/A/Mexico/MEX-29/2007|EPI_ISL_2585205|2007-02-16
hRSV/A/USA/LA2-49/2013|EPI_ISL_2588349|2013-01-10
hRSV/A/Kenya/KILIFI-WGS-1284-02-01/2015|EPI_ISL_2595653|2015-01-02

hRSV/A/USA/UAMS-DID-009/2016|EPI_ISL_2595700|2016-01-23
hRSV/A/England/104800018/2010|EPI_ISL_6494935|2010-11-25
**RSV-B Reference**
hRSV/B/Argentina/BA-HNRG-014/2014|EPI_ISL_1074215|2014-05-20
hRSV/B/USA/TX-79362/2005|EPI_ISL_15751944|2005
hRSV/B/Kenya/58081/2007|EPI_ISL_15751949|2007
hRSV/B/Brazil/un-38663/2010|EPI_ISL_15751954|2010
hRSV/B/Brazil/un-38219/2009|EPI_ISL_15751992|2009
hRSV/B/Brazil/un-37940/2008|EPI_ISL_15752003|2008
hRSV/B/Germany/BE-08-00915/2008|EPI_ISL_18005907|2008-01-29
hRSV/B/Germany/BY-04-02666/2004|EPI_ISL_18005929|2004-04-16
hRSV/B/Germany/NI-09-03432/2009|EPI_ISL_18005947|2009-02-10
hRSV/B/Germany/BE-00-02068/2000|EPI_ISL_18005950|2000-02-07
hRSV/B/Germany/un-RKI-485/2003|EPI_ISL_18891883|2003-12-08
hRSV/B/Germany/un-RKI-329/2000|EPI_ISL_18891886|2000-12-14
hRSV/B/Germany/un-RKI-3264/2006|EPI_ISL_18892065|2006-04-25
hRSV/B/Germany/un-RKI-2512/2001|EPI_ISL_18892073|2001-03-12
hRSV/B/Hong_Kong/PHLC-V11-2224258/2011|EPI_ISL_19813066|2011-03-20
hRSV/B/Netherlands/02-028215/2002|EPI_ISL_2544114|2002-10-21
hRSV/B/Argentina/187/2006|EPI_ISL_2544180|2006-07-25
hRSV/B/Japan/B-Sendai-240-10/2010|EPI_ISL_2558895|2010
hRSV/B/Jordan/JOR-C2195/2011|EPI_ISL_2575493|2011-03-10
hRSV/B/Kenya/Kilifi-HH-4201-06-Feb/2010|EPI_ISL_2577404|2010-02-06
hRSV/B/United_Kingdom/313/2012|EPI_ISL_2577421|2012-12-21
hRSV/B/Jordan/JOR-A0650/2012|EPI_ISL_2577698|2012-12-20
hRSV/B/Philippines/TB5-CA-14-0377-2-1/2014|EPI_ISL_2578157|2014-07-31
hRSV/B/Netherlands/05-001965/2005|EPI_ISL_2584598|2005-01-05
hRSV/B/Mexico/MEX-20/2004|EPI_ISL_2584621|2004-10-18
hRSV/B/Kenya/Kilifi-9697-7/2004|EPI_ISL_2584626|2004-12-25
hRSV/B/Netherlands/03-034613/2003|EPI_ISL_2584634|2003-11-25
hRSV/B/USA/TH-10447/2013|EPI_ISL_2584905|2013-10-22
hRSV/B/New_Zealand/NZL-LJRSV50/2013|EPI_ISL_2585068|2013-10-22
hRSV/B/Peru/PER-FLU5540/2007|EPI_ISL_2585144|2007-01-26
hRSV/B/United_Kingdom/B05/2005|EPI_ISL_2585146|2005-01-06
hRSV/B/Italy/LOM-129/2009|EPI_ISL_2588656|2009-02-18
hRSV/B/England/100580129/2010|EPI_ISL_6494929|2010-02-04
hRSV/B/England/125280313/2012|EPI_ISL_6494978|2012-12-21
hRSV/B/Morocco/295-16/2016|EPI_ISL_15120699|2016-01-20
hRSV/B/Austria/MUW1119688/2019|EPI_ISL_16533855|2019-01-25
hRSV/B/Mozambique/MAP-IR0529/2015|EPI_ISL_17597891|2015-02-27
hRSV/B/Germany/RP-19-01738/2019|EPI_ISL_18005838|2019-01-24

hRSV/B/United_Kingdom/UMC-26428/2019|EPI_ISL_18061844|2019-01-24
hRSV/B/Spain/CT-HUVH-765080/2018|EPI_ISL_18682239|2018-01-25
hRSV/B/Spain/CT-HUVH-141989/2015|EPI_ISL_18682260|2015-01-02
hRSV/B/Netherlands/un-UMC-310101065/2018|EPI_ISL_19048177|2018-01-25
hRSV/B/New_Zealand/IESR-HB0181/2017|EPI_ISL_19206973|2017-05-30
hRSV/B/Beijing/BCDC-224/2015|EPI_ISL_19542547|2015-01-06
hRSV/B/China/ZJ-CDC-17S029/2017|EPI_ISL_19814934|2017-02-21
hRSV/B/Nicaragua/NIC-IIIn-16-0118-01NT/2016|EPI_ISL_2577441|2016-08-25
hRSV/B/Kenya/KHDSS-78-06-JUN/2017|EPI_ISL_2584674|2017-02-20
hRSV/B/Kenya/KHDSS-68-04-JUN/2016|EPI_ISL_2585059|2016-01-27
hRSV/B/Brazil/RS-FIOCRUZ-68492/2021|EPI_ISL_15895023|2021-04-13
hRSV/B/Finland/UMC-358103029/2020|EPI_ISL_19048001|2020-02-14
hRSV/B/Scotland/IEE-LO258/2020|EPI_ISL_19442608|2020-01-01
hRSV/B/Beijing/BCDC-R0976/2020|EPI_ISL_19542733|2020-01-01
hRSV/B/China/WH-WIV-0416n2/2021|EPI_ISL_19888417|2021-04-16
hRSV/B/USA/NY-CH43II/1990|EPI_ISL_15771617|1990
hRSV/B/USA/NY-CH59III/1995|EPI_ISL_15771618|1995
hRSV/B/Japan/KU-002/1997|EPI_ISL_17782656|1997-12-01
hRSV/B/United_Kingdom/GLS-NU613/1997|EPI_ISL_19556972|1997
hRSV/B/USA/9320/1977|EPI_ISL_2544151|1977
hRSV/B/USA/79E-159-01-1979/1979|EPI_ISL_2544153|1979
hRSV/B/USA/B-WaDC-18537-1962-WGS/1962|EPI_ISL_2559012|1962
hRSV/B/USA/95I-016A-01-01/1995|EPI_ISL_2584601|1995
hRSV/B/USA/95I-100A-01/1995|EPI_ISL_2584606|1995
hRSV/B/USA/96E-113-01/1996|EPI_ISL_2584610|1996
hRSV/B/USA/96I-097A-01/1996|EPI_ISL_2584615|1996
hRSV/B/USA/87I-028A-01/1987|EPI_ISL_2584684|1987
hRSV/B/USA/89P-004-01/1989|EPI_ISL_2584753|1989
hRSV/B/Italy/LOM-01000074/2023|EPI_ISL_18329476|2023-01-01
hRSV/B/Bulgaria/s121/2023|EPI_ISL_18447481|2023-01-01
hRSV/B/England/UKHSA_RVU_4000306/2023|EPI_ISL_19276849|2023-01-01
hRSV/B/Canada/ON-KHS-00877-v1/2024|EPI_ISL_19321121|2024-01-01
hRSV/B/Philippines/RITM0012/2024|EPI_ISL_19701258|2024-01-01
hRSV/B/Australia/TAS-HHR-054937/2024|EPI_ISL_20195438|2024-01-01
hRSV/B/Spain/CHUVI-19491616/2022|EPI_ISL_14084081|2022-01-03
hRSV/B/Beijing/BCDC-R0126/2022|EPI_ISL_19542721|2022-01-06
hRSV/B/Portugal/INSA-32/2021|EPI_ISL_20093630|2022-01-04
