# Supplementary material for: Whole-Genome Phylodynamic Analysis of Respiratory Syncytial Virus—Maryland, USA, 2018–2024
Source: Viruses. 2026 Mar 7;18(3):331. doi: 10.3390/v18030331 (PMC13030589; doi:10.3390/v18030331)
Supplement: Supplementary file 1 [file viruses-18-00331-s001.zip › Supplementary Table S3.pdf]

Supplementary Table S3. Clock model comparison with marginal likelihood and ESS on the whole-genome of RSV-A and RSV-B sequences.

RSV-A		
Model	Marginal L Estimate	Sum(ESS)
Strict	-65764.7643	1006.4102
Uncorrelated exponential	-68869.2444	760.7449
Uncorrelated lognormal	-33385.0376	1102.5804
Random local	-67567.2698	782.6262
Best RSV-A Clock: Uncorrelated lognormal		
RSV-B		
Model	Marginal L Estimate	Sum(ESS)
Strict	-57820.3912	1967.0982
Uncorrelated exponential	-42264.7523	1218.6211
Uncorrelated lognormal	-42254.7748	1094.2600
Random local	-73364.5727	1254.2607
Best RSV-B Clock: Uncorrelated lognormal		
