# Supplementary material for: Whole-Genome Phylodynamic Analysis of Respiratory Syncytial Virus—Maryland, USA, 2018–2024
Source: Viruses. 2026 Mar 7;18(3):331. doi: 10.3390/v18030331 (PMC13030589; doi:10.3390/v18030331)
Supplement: Supplementary file 1 [file viruses-18-00331-s001.zip › Supplementary Table S4.pdf]

Supplementary Table 4. Gene-specific nucleotide and amino acid entropy in RSV-A and RSV-B.

Gene	Metric	RSV-A (95% CI)	RSV-B (95% CI)	p-value	q-value
F	Nucleotide	0.0159 (0.0128-0.0192)	0.0138 (0.0111-0.0168)	5.31E-06	9.73E-06
G	Nucleotide	0.0426 (0.0352-0.0503)	0.0349 (0.0274-0.0430)	7.73E-05	0.000121
L	Nucleotide	0.0108 (0.0092-0.0126)	0.0144 (0.0128-0.0160)	5.24E-110	5.76E-109
M	Nucleotide	0.0186 (0.0135-0.0242)	0.0143 (0.0098-0.0196)	0.00106	0.00146
M2-1	Nucleotide	0.0184 (0.0138-0.0235)	0.0090 (0.0063-0.0124)	2.05E-18	5.64E-18
M2-2	Nucleotide	0.0392 (0.0320-0.0463)	0.0166 (0.0082-0.0269)	4.93E-50	2.71E-49
N	Nucleotide	0.0182 (0.0143-0.0224)	0.0094 (0.0072-0.0119)	7.87E-28	2.88E-27
NS1	Nucleotide	0.0146 (0.0086-0.0214)	0.0077 (0.0050-0.0110)	0.0344	0.042
NS2	Nucleotide	0.0192 (0.0119-0.0274)	0.0156 (0.0108-0.0214)	0.771	0.771
P	Nucleotide	0.0186 (0.0137-0.0240)	0.0142 (0.0104-0.0183)	4.73E-11	1.04E-10
SH	Nucleotide	0.0264 (0.0140-0.0400)	0.0154 (0.0098-0.0213)	0.678	0.745
F	Amino acid	0.0075 (0.0044-0.0113)	0.0096 (0.0051-0.0152)	0.0872	0.12
G	Amino acid	0.0717 (0.0545-0.0896)	0.0593 (0.0436-0.0763)	0.0192	0.0352
L	Amino acid	0.0121 (0.0096-0.0149)	0.0087 (0.0071-0.0105)	6.41E-11	7.05E-10
M	Amino acid	0.0070 (0.0019-0.0138)	0.0013 (0.0004-0.0022)	0.0418	0.0656
M2-1	Amino acid	0.0095 (0.0053-0.0147)	0.0045 (0.0018-0.0081)	0.00559	0.0154
M2-2	Amino acid	0.0295 (0.0167-0.0449)	0.0369 (0.0134-0.0662)	0.0102	0.0224
N	Amino acid	0.0046 (0.0028-0.0069)	0.0019 (0.0010-0.0030)	4.66E-07	1.71E-06
NS1	Amino acid	0.0026 (0.0005-0.0053)	0.0043 (0.0020-0.0069)	0.699	0.768
NS2	Amino acid	0.0109 (0.0039-0.0212)	0.0126 (0.0045-0.0242)	0.88	0.88
P	Amino acid	0.0093 (0.0052-0.0144)	0.0042 (0.0014-0.0078)	2.19E-07	1.21E-06
SH	Amino acid	0.0208 (0.0014-0.0490)	0.0204 (0.0096-0.0340)	0.179	0.219
