# Supplementary material for: Whole-Genome Phylodynamic Analysis of Respiratory Syncytial Virus—Maryland, USA, 2018–2024
Source: Viruses. 2026 Mar 7;18(3):331. doi: 10.3390/v18030331 (PMC13030589; doi:10.3390/v18030331)
Supplement: Supplementary file 1 [file viruses-18-00331-s001.zip › Supplementary Table S5.pdf]

Supplementary Table 5. Distribution of RSV Subtypes and Clades from 2018 to 2024 Cohort

Year	Subtype	Total	Clade	Number of Samples (% cohort)
2018	A	16	A.D	10 (62.5)
			A.D.1	3 (18.8)
			A.D.3	2 (12.5)
			A.D.5	1 (6.2)
	B	40	B.D.4.1	8 (20)
			B.D.4.1.1	32 (80)
2019	A	74	A.D	2 (2.7)
			A.D.1	61 (82.4)
			A.D.3	3 (4.1)
			A.D.5	6 (8.1)
			A.D.5.1	2 (2.7)
	B	5	B.D.4.1.1	5 (100)
2020	A	54	A.D	2 (3.7)
			A.D.1	43 (79.6)
			A.D.1.1	1 (1.8)
			A.D.2.2	2 (3.7)
			A.D.5	5 (9.2)

			A.D.5.1	1 (3.7)
	B	9	B.D.4.1.1	9 (100)
2022	A	52	A.D.1	8 (15.4)
			A.D.1.5	4 (7.7)
			A.D.1.7	2 (3.8)
			A.D.1.8	5 (9.6)
			A.D.3	2 (3.8)
			A.D.3.2	3 (5.8)
			A.D.5	2 (3.8)
			A.D.5.1	2 (3.8)
			A.D.5.2	24 (46.2)
	B	13	B.D.E.1	12 (92.3)
			B.D.4.1.1	1 (7.7)
2023	A	19	A.D.1	1 (5.3)
			A.D.1.5	4 (21.1)
			A.D.1.6	3 (15.8)
			A.D.3	2 (10.5)
			A.D.5.1	2 (10.5)
			A.D.5.2	7 (36.8)

	B	57	B.D.E.1	53 (93.0)
			B.D.E.1.4	1 (1.8)
			B.D.4.1.1	3 (5.3)
2024	A	95	A.D	1 (1.1)
			A.D.1	4 (4.2)
			A.D.1.5	13 (13.7)
			A.D.1.6	62 (65.3)
			A.D.1.8	2 (2.1)
			A.D.3	2 (2.1)
			A.D.3.2	1 (1.1)
			A.D.3.3	1 (1.1)
			A.D.5.2	9 (9.5)
	B	17	B.D.E.1	16 (94.1)
			B.D.E.1.1	1 (5.9)
