# Supplementary material for: Whole-Genome Phylodynamic Analysis of Respiratory Syncytial Virus—Maryland, USA, 2018–2024
Source: Viruses. 2026 Mar 7;18(3):331. doi: 10.3390/v18030331 (PMC13030589; doi:10.3390/v18030331)
Supplement: Supplementary file 1 [file viruses-18-00331-s001.zip › Supplementary Table S6.pdf]

Supplementary Table S6. GISAID Accession Numbers for RSV-A and RSV-B Sequences

**RSV strains**	**Accession ID**
hRSV/A/USA/MD-JHRSV1007/2023	EPI_ISL_19884058
hRSV/A/USA/MD-JHRSV1011/2023	EPI_ISL_19884059
hRSV/A/USA/MD-JHRSV1020/2023	EPI_ISL_19884060
hRSV/A/USA/MD-JHRSV461/2019	EPI_ISL_19883841
hRSV/A/USA/MD-JHRSV462/2019	EPI_ISL_19883842
hRSV/A/USA/MD-JHRSV464/2019	EPI_ISL_19883843
hRSV/A/USA/MD-JHRSV465/2019	EPI_ISL_19883844
hRSV/A/USA/MD-JHRSV467/2019	EPI_ISL_19883845
hRSV/A/USA/MD-JHRSV468/2019	EPI_ISL_19883846
hRSV/A/USA/MD-JHRSV470/2019	EPI_ISL_19883847
hRSV/A/USA/MD-JHRSV471/2019	EPI_ISL_19883848
hRSV/A/USA/MD-JHRSV473/2019	EPI_ISL_19883849
hRSV/A/USA/MD-JHRSV474/2019	EPI_ISL_19883850
hRSV/A/USA/MD-JHRSV475/2019	EPI_ISL_19883851
hRSV/A/USA/MD-JHRSV477/2019	EPI_ISL_19883852
hRSV/A/USA/MD-JHRSV478/2019	EPI_ISL_19883853
hRSV/A/USA/MD-JHRSV479/2019	EPI_ISL_19883854
hRSV/A/USA/MD-JHRSV480/2019	EPI_ISL_19883855
hRSV/A/USA/MD-JHRSV481/2019	EPI_ISL_19883856
hRSV/A/USA/MD-JHRSV483/2019	EPI_ISL_19883857
hRSV/A/USA/MD-JHRSV484/2019	EPI_ISL_19883858
hRSV/A/USA/MD-JHRSV486/2019	EPI_ISL_19883859
hRSV/A/USA/MD-JHRSV487/2019	EPI_ISL_19883860
hRSV/A/USA/MD-JHRSV490/2019	EPI_ISL_19883861
hRSV/A/USA/MD-JHRSV491/2019	EPI_ISL_19883862
hRSV/A/USA/MD-JHRSV492/2019	EPI_ISL_19883863
hRSV/A/USA/MD-JHRSV493/2019	EPI_ISL_19883864
hRSV/A/USA/MD-JHRSV494/2019	EPI_ISL_19883865
hRSV/A/USA/MD-JHRSV495/2019	EPI_ISL_19883866
hRSV/A/USA/MD-JHRSV496/2019	EPI_ISL_19883867
hRSV/A/USA/MD-JHRSV498/2019	EPI_ISL_19883868
hRSV/A/USA/MD-JHRSV499/2019	EPI_ISL_19883869
hRSV/A/USA/MD-JHRSV500/2019	EPI_ISL_19883870
hRSV/A/USA/MD-JHRSV501/2019	EPI_ISL_19883871
hRSV/A/USA/MD-JHRSV502/2019	EPI_ISL_19883872
hRSV/A/USA/MD-JHRSV503/2019	EPI_ISL_19883873
hRSV/A/USA/MD-JHRSV504/2019	EPI_ISL_19883874
hRSV/A/USA/MD-JHRSV505/2019	EPI_ISL_19883875
hRSV/A/USA/MD-JHRSV508/2019	EPI_ISL_19883876

hRSV/A/USA/MD-JHRSV509/2019	EPI_ISL_19883877
hRSV/A/USA/MD-JHRSV510/2019	EPI_ISL_19883878
hRSV/A/USA/MD-JHRSV512/2019	EPI_ISL_19883879
hRSV/A/USA/MD-JHRSV513/2019	EPI_ISL_19883880
hRSV/A/USA/MD-JHRSV515/2019	EPI_ISL_19883881
hRSV/A/USA/MD-JHRSV516/2019	EPI_ISL_19883882
hRSV/A/USA/MD-JHRSV517/2019	EPI_ISL_19883883
hRSV/A/USA/MD-JHRSV518/2019	EPI_ISL_19883884
hRSV/A/USA/MD-JHRSV520/2019	EPI_ISL_19883885
hRSV/A/USA/MD-JHRSV521/2019	EPI_ISL_19883886
hRSV/A/USA/MD-JHRSV522/2019	EPI_ISL_19883887
hRSV/A/USA/MD-JHRSV526/2019	EPI_ISL_19883888
hRSV/A/USA/MD-JHRSV527/2019	EPI_ISL_19883889
hRSV/A/USA/MD-JHRSV528/2019	EPI_ISL_19883890
hRSV/A/USA/MD-JHRSV529/2019	EPI_ISL_19883891
hRSV/A/USA/MD-JHRSV530/2019	EPI_ISL_19883892
hRSV/A/USA/MD-JHRSV531/2019	EPI_ISL_19883893
hRSV/A/USA/MD-JHRSV532/2019	EPI_ISL_19883894
hRSV/A/USA/MD-JHRSV533/2019	EPI_ISL_19883895
hRSV/A/USA/MD-JHRSV534/2019	EPI_ISL_19883896
hRSV/A/USA/MD-JHRSV535/2019	EPI_ISL_19883897
hRSV/A/USA/MD-JHRSV536/2019	EPI_ISL_19883898
hRSV/A/USA/MD-JHRSV537/2019	EPI_ISL_19883899
hRSV/A/USA/MD-JHRSV538/2019	EPI_ISL_19883900
hRSV/A/USA/MD-JHRSV539/2019	EPI_ISL_19883901
hRSV/A/USA/MD-JHRSV540/2019	EPI_ISL_19883902
hRSV/A/USA/MD-JHRSV541/2019	EPI_ISL_19883903
hRSV/A/USA/MD-JHRSV542/2019	EPI_ISL_19883904
hRSV/A/USA/MD-JHRSV544/2019	EPI_ISL_19883905
hRSV/A/USA/MD-JHRSV545/2019	EPI_ISL_19883906
hRSV/A/USA/MD-JHRSV547/2019	EPI_ISL_19883907
hRSV/A/USA/MD-JHRSV548/2019	EPI_ISL_19883908
hRSV/A/USA/MD-JHRSV549/2019	EPI_ISL_19883909
hRSV/A/USA/MD-JHRSV550/2019	EPI_ISL_19883910
hRSV/A/USA/MD-JHRSV552/2019	EPI_ISL_19883911
hRSV/A/USA/MD-JHRSV553/2019	EPI_ISL_19883912
hRSV/A/USA/MD-JHRSV554/2019	EPI_ISL_19883913
hRSV/A/USA/MD-JHRSV555/2019	EPI_ISL_19883914
hRSV/A/USA/MD-JHRSV583/2018	EPI_ISL_19883915
hRSV/A/USA/MD-JHRSV585/2018	EPI_ISL_19883916
hRSV/A/USA/MD-JHRSV593/2018	EPI_ISL_19883917

hRSV/A/USA/MD-JHRSV605/2018	EPI_ISL_19883918
hRSV/A/USA/MD-JHRSV606/2018	EPI_ISL_19883919
hRSV/A/USA/MD-JHRSV607/2018	EPI_ISL_19883920
hRSV/A/USA/MD-JHRSV614/2018	EPI_ISL_19883921
hRSV/A/USA/MD-JHRSV620/2018	EPI_ISL_19883922
hRSV/A/USA/MD-JHRSV622/2018	EPI_ISL_19883923
hRSV/A/USA/MD-JHRSV631/2018	EPI_ISL_19883924
hRSV/A/USA/MD-JHRSV632/2018	EPI_ISL_19883925
hRSV/A/USA/MD-JHRSV633/2018	EPI_ISL_19883926
hRSV/A/USA/MD-JHRSV634/2018	EPI_ISL_19883927
hRSV/A/USA/MD-JHRSV635/2018	EPI_ISL_19883928
hRSV/A/USA/MD-JHRSV644/2018	EPI_ISL_19883929
hRSV/A/USA/MD-JHRSV646/2018	EPI_ISL_19883930
hRSV/A/USA/MD-JHRSV647/2018	EPI_ISL_19883931
hRSV/A/USA/MD-JHRSV651/2022	EPI_ISL_19883932
hRSV/A/USA/MD-JHRSV652/2022	EPI_ISL_19883933
hRSV/A/USA/MD-JHRSV653/2022	EPI_ISL_19883934
hRSV/A/USA/MD-JHRSV655/2022	EPI_ISL_19883935
hRSV/A/USA/MD-JHRSV656/2022	EPI_ISL_19883936
hRSV/A/USA/MD-JHRSV658/2022	EPI_ISL_19883937
hRSV/A/USA/MD-JHRSV659/2022	EPI_ISL_19883938
hRSV/A/USA/MD-JHRSV665/2022	EPI_ISL_19883939
hRSV/A/USA/MD-JHRSV668/2022	EPI_ISL_19883940
hRSV/A/USA/MD-JHRSV669/2022	EPI_ISL_19883941
hRSV/A/USA/MD-JHRSV670/2022	EPI_ISL_19883942
hRSV/A/USA/MD-JHRSV673/2022	EPI_ISL_19883943
hRSV/A/USA/MD-JHRSV674/2022	EPI_ISL_19883944
hRSV/A/USA/MD-JHRSV675/2022	EPI_ISL_19883945
hRSV/A/USA/MD-JHRSV676/2022	EPI_ISL_19883946
hRSV/A/USA/MD-JHRSV678/2022	EPI_ISL_19883947
hRSV/A/USA/MD-JHRSV679/2022	EPI_ISL_19883948
hRSV/A/USA/MD-JHRSV680/2022	EPI_ISL_19883949
hRSV/A/USA/MD-JHRSV681/2022	EPI_ISL_19883950
hRSV/A/USA/MD-JHRSV682/2022	EPI_ISL_19883951
hRSV/A/USA/MD-JHRSV683/2022	EPI_ISL_19883952
hRSV/A/USA/MD-JHRSV684/2022	EPI_ISL_19883953
hRSV/A/USA/MD-JHRSV685/2022	EPI_ISL_19883954
hRSV/A/USA/MD-JHRSV687/2022	EPI_ISL_19883955
hRSV/A/USA/MD-JHRSV688/2022	EPI_ISL_19883956
hRSV/A/USA/MD-JHRSV691/2022	EPI_ISL_19883957
hRSV/A/USA/MD-JHRSV692/2022	EPI_ISL_19883958

hRSV/A/USA/MD-JHRSV694/2022	EPI_ISL_19883959
hRSV/A/USA/MD-JHRSV695/2022	EPI_ISL_19883960
hRSV/A/USA/MD-JHRSV696/2022	EPI_ISL_19883961
hRSV/A/USA/MD-JHRSV698/2022	EPI_ISL_19883962
hRSV/A/USA/MD-JHRSV699/2022	EPI_ISL_19883963
hRSV/A/USA/MD-JHRSV701/2022	EPI_ISL_19883964
hRSV/A/USA/MD-JHRSV702/2022	EPI_ISL_19883965
hRSV/A/USA/MD-JHRSV703/2022	EPI_ISL_19883966
hRSV/A/USA/MD-JHRSV704/2022	EPI_ISL_19883967
hRSV/A/USA/MD-JHRSV705/2022	EPI_ISL_19883968
hRSV/A/USA/MD-JHRSV707/2022	EPI_ISL_19883969
hRSV/A/USA/MD-JHRSV708/2022	EPI_ISL_19883970
hRSV/A/USA/MD-JHRSV710/2022	EPI_ISL_19883971
hRSV/A/USA/MD-JHRSV711/2022	EPI_ISL_19883972
hRSV/A/USA/MD-JHRSV713/2022	EPI_ISL_19883973
hRSV/A/USA/MD-JHRSV714/2022	EPI_ISL_19883974
hRSV/A/USA/MD-JHRSV718/2022	EPI_ISL_19883975
hRSV/A/USA/MD-JHRSV719/2022	EPI_ISL_19883976
hRSV/A/USA/MD-JHRSV720/2022	EPI_ISL_19883977
hRSV/A/USA/MD-JHRSV721/2022	EPI_ISL_19883978
hRSV/A/USA/MD-JHRSV723/2022	EPI_ISL_19883979
hRSV/A/USA/MD-JHRSV725/2022	EPI_ISL_19883980
hRSV/A/USA/MD-JHRSV726/2022	EPI_ISL_19883981
hRSV/A/USA/MD-JHRSV730/2022	EPI_ISL_19883982
hRSV/A/USA/MD-JHRSV732/2022	EPI_ISL_19883983
hRSV/A/USA/MD-JHRSV733/2022	EPI_ISL_19883984
hRSV/A/USA/MD-JHRSV739/2020	EPI_ISL_19883985
hRSV/A/USA/MD-JHRSV740/2020	EPI_ISL_19883986
hRSV/A/USA/MD-JHRSV745/2020	EPI_ISL_19883987
hRSV/A/USA/MD-JHRSV746/2020	EPI_ISL_19883988
hRSV/A/USA/MD-JHRSV747/2020	EPI_ISL_19883989
hRSV/A/USA/MD-JHRSV750/2020	EPI_ISL_19883990
hRSV/A/USA/MD-JHRSV751/2020	EPI_ISL_19883991
hRSV/A/USA/MD-JHRSV752/2020	EPI_ISL_19883992
hRSV/A/USA/MD-JHRSV754/2020	EPI_ISL_19883993
hRSV/A/USA/MD-JHRSV756/2020	EPI_ISL_19883994
hRSV/A/USA/MD-JHRSV757/2020	EPI_ISL_19883995
hRSV/A/USA/MD-JHRSV758/2020	EPI_ISL_19883996
hRSV/A/USA/MD-JHRSV762/2020	EPI_ISL_19883997
hRSV/A/USA/MD-JHRSV763/2020	EPI_ISL_19883998
hRSV/A/USA/MD-JHRSV764/2020	EPI_ISL_19883999

hRSV/A/USA/MD-JHRSV767/2020	EPI_ISL_19884000
hRSV/A/USA/MD-JHRSV770/2020	EPI_ISL_19884001
hRSV/A/USA/MD-JHRSV772/2020	EPI_ISL_19884002
hRSV/A/USA/MD-JHRSV776/2020	EPI_ISL_19884003
hRSV/A/USA/MD-JHRSV778/2020	EPI_ISL_19884004
hRSV/A/USA/MD-JHRSV779/2020	EPI_ISL_19884005
hRSV/A/USA/MD-JHRSV780/2020	EPI_ISL_19884006
hRSV/A/USA/MD-JHRSV784/2020	EPI_ISL_19884007
hRSV/A/USA/MD-JHRSV786/2020	EPI_ISL_19884008
hRSV/A/USA/MD-JHRSV787/2020	EPI_ISL_19884009
hRSV/A/USA/MD-JHRSV788/2020	EPI_ISL_19884010
hRSV/A/USA/MD-JHRSV790/2020	EPI_ISL_19884011
hRSV/A/USA/MD-JHRSV791/2020	EPI_ISL_19884012
hRSV/A/USA/MD-JHRSV792/2020	EPI_ISL_19884013
hRSV/A/USA/MD-JHRSV795/2020	EPI_ISL_19884014
hRSV/A/USA/MD-JHRSV796/2020	EPI_ISL_19884015
hRSV/A/USA/MD-JHRSV799/2020	EPI_ISL_19884016
hRSV/A/USA/MD-JHRSV801/2020	EPI_ISL_19884017
hRSV/A/USA/MD-JHRSV802/2020	EPI_ISL_19884018
hRSV/A/USA/MD-JHRSV803/2020	EPI_ISL_19884019
hRSV/A/USA/MD-JHRSV804/2020	EPI_ISL_19884020
hRSV/A/USA/MD-JHRSV805/2020	EPI_ISL_19884021
hRSV/A/USA/MD-JHRSV806/2020	EPI_ISL_19884022
hRSV/A/USA/MD-JHRSV807/2020	EPI_ISL_19884023
hRSV/A/USA/MD-JHRSV808/2020	EPI_ISL_19884024
hRSV/A/USA/MD-JHRSV809/2020	EPI_ISL_19884025
hRSV/A/USA/MD-JHRSV810/2020	EPI_ISL_19884026
hRSV/A/USA/MD-JHRSV811/2020	EPI_ISL_19884027
hRSV/A/USA/MD-JHRSV812/2020	EPI_ISL_19884028
hRSV/A/USA/MD-JHRSV813/2020	EPI_ISL_19884029
hRSV/A/USA/MD-JHRSV818/2020	EPI_ISL_19884030
hRSV/A/USA/MD-JHRSV819/2020	EPI_ISL_19884031
hRSV/A/USA/MD-JHRSV823/2020	EPI_ISL_19884032
hRSV/A/USA/MD-JHRSV824/2020	EPI_ISL_19884033
hRSV/A/USA/MD-JHRSV826/2020	EPI_ISL_19884034
hRSV/A/USA/MD-JHRSV827/2020	EPI_ISL_19884035
hRSV/A/USA/MD-JHRSV828/2020	EPI_ISL_19884036
hRSV/A/USA/MD-JHRSV829/2020	EPI_ISL_19884037
hRSV/A/USA/MD-JHRSV830/2020	EPI_ISL_19884038
hRSV/A/USA/MD-JHRSV832/2020	EPI_ISL_19884039
hRSV/A/USA/MD-JHRSV943/2023	EPI_ISL_19884040

hRSV/A/USA/MD-JHRSV951/2023	EPI_ISL_19884041
hRSV/A/USA/MD-JHRSV962/2023	EPI_ISL_19884042
hRSV/A/USA/MD-JHRSV963/2023	EPI_ISL_19884043
hRSV/A/USA/MD-JHRSV965/2023	EPI_ISL_19884044
hRSV/A/USA/MD-JHRSV970/2023	EPI_ISL_19884045
hRSV/A/USA/MD-JHRSV975/2023	EPI_ISL_19884046
hRSV/A/USA/MD-JHRSV976/2023	EPI_ISL_19884047
hRSV/A/USA/MD-JHRSV977/2023	EPI_ISL_19884048
hRSV/A/USA/MD-JHRSV978/2023	EPI_ISL_19884049
hRSV/A/USA/MD-JHRSV981/2023	EPI_ISL_19884050
hRSV/A/USA/MD-JHRSV982/2023	EPI_ISL_19884051
hRSV/A/USA/MD-JHRSV984/2023	EPI_ISL_19884052
hRSV/A/USA/MD-JHRSV985/2023	EPI_ISL_19884053
hRSV/A/USA/MD-JHRSV986/2023	EPI_ISL_19884054
hRSV/A/USA/MD-JHRSV987/2023	EPI_ISL_19884055
hRSV/A/USA/MD-JHRSV988/2023	EPI_ISL_19884056
hRSV/A/USA/MD-JHRSV992/2023	EPI_ISL_19884057
hRSV/A/USA/MD-JHSOM103/2024	EPI_ISL_19818901
hRSV/A/USA/MD-JHSOM106/2024	EPI_ISL_19818904
hRSV/A/USA/MD-JHSOM110/2024	EPI_ISL_19818907
hRSV/A/USA/MD-JHSOM119/2024	EPI_ISL_19818915
hRSV/A/USA/MD-JHSOM121/2024	EPI_ISL_19818916
hRSV/A/USA/MD-JHSOM125/2024	EPI_ISL_19818920
hRSV/A/USA/MD-JHSOM132/2024	EPI_ISL_19818927
hRSV/A/USA/MD-JHSOM136/2024	EPI_ISL_19818931
hRSV/A/USA/MD-JHSOM140/2024	EPI_ISL_19818935
hRSV/A/USA/MD-JHSOM166/2024	EPI_ISL_19818951
hRSV/A/USA/MD-JHSOM168/2024	EPI_ISL_19818953
hRSV/A/USA/MD-JHSOM169/2024	EPI_ISL_19818954
hRSV/A/USA/MD-JHSOM170/2024	EPI_ISL_19818955
hRSV/A/USA/MD-JHSOM171/2024	EPI_ISL_19818956
hRSV/A/USA/MD-JHSOM173/2024	EPI_ISL_19818958
hRSV/A/USA/MD-JHSOM177/2024	EPI_ISL_19818962
hRSV/A/USA/MD-JHSOM178/2024	EPI_ISL_19818963
hRSV/A/USA/MD-JHSOM182/2024	EPI_ISL_19818965
hRSV/A/USA/MD-JHSOM187/2024	EPI_ISL_19818970
hRSV/A/USA/MD-JHSOM189/2024	EPI_ISL_19818972
hRSV/A/USA/MD-JHSOM195/2024	EPI_ISL_19818976
hRSV/A/USA/MD-JHSOM206/2024	EPI_ISL_19818987
hRSV/A/USA/MD-JHSOM212/2024	EPI_ISL_19818991
hRSV/A/USA/MD-JHSOM223/2024	EPI_ISL_19818996

hRSV/A/USA/MD-JHSOM237/2024	EPI_ISL_19819005
hRSV/A/USA/MD-JHSOM239/2024	EPI_ISL_19819007
hRSV/A/USA/MD-JHSOM240/2024	EPI_ISL_19819008
hRSV/A/USA/MD-JHSOM242/2024	EPI_ISL_19819010
hRSV/A/USA/MD-JHSOM245/2024	EPI_ISL_19819012
hRSV/A/USA/MD-JHSOM246/2024	EPI_ISL_19819013
hRSV/A/USA/MD-JHSOM248/2024	EPI_ISL_19819014
hRSV/A/USA/MD-JHSOM254/2024	EPI_ISL_19819018
hRSV/A/USA/MD-JHSOM259/2024	EPI_ISL_19819022
hRSV/A/USA/MD-JHSOM262/2024	EPI_ISL_19819023
hRSV/A/USA/MD-JHSOM272/2024	EPI_ISL_19819029
hRSV/A/USA/MD-JHSOM275/2024	EPI_ISL_19819031
hRSV/A/USA/MD-JHSOM277/2024	EPI_ISL_19819032
hRSV/A/USA/MD-JHSOM279/2024	EPI_ISL_19819034
hRSV/A/USA/MD-JHSOM283/2024	EPI_ISL_19819037
hRSV/A/USA/MD-JHSOM292/2024	EPI_ISL_19819043
hRSV/A/USA/MD-JHSOM297/2024	EPI_ISL_19819047
hRSV/A/USA/MD-JHSOM302/2024	EPI_ISL_19819051
hRSV/A/USA/MD-JHSOM304/2024	EPI_ISL_19819053
hRSV/A/USA/MD-JHSOM306/2024	EPI_ISL_19819055
hRSV/A/USA/MD-JHSOM307/2024	EPI_ISL_19819056
hRSV/A/USA/MD-JHSOM318/2024	EPI_ISL_19819064
hRSV/A/USA/MD-JHSOM323/2024	EPI_ISL_19819068
hRSV/A/USA/MD-JHSOM324/2024	EPI_ISL_19819069
hRSV/A/USA/MD-JHSOM325/2024	EPI_ISL_19819070
hRSV/A/USA/MD-JHSOM328/2024	EPI_ISL_19819073
hRSV/A/USA/MD-JHSOM331/2024	EPI_ISL_19819075
hRSV/A/USA/MD-JHSOM352/2024	EPI_ISL_19819093
hRSV/A/USA/MD-JHSOM353/2024	EPI_ISL_19819094
hRSV/A/USA/MD-JHSOM355/2024	EPI_ISL_19819096
hRSV/A/USA/MD-JHSOM357/2024	EPI_ISL_19819098
hRSV/A/USA/MD-JHSOM359/2024	EPI_ISL_19819100
hRSV/A/USA/MD-JHSOM361/2024	EPI_ISL_19819102
hRSV/A/USA/MD-JHSOM363/2024	EPI_ISL_19819104
hRSV/A/USA/MD-JHSOM374/2024	EPI_ISL_19819110
hRSV/A/USA/MD-JHSOM380/2024	EPI_ISL_19819115
hRSV/A/USA/MD-JHSOM382/2024	EPI_ISL_19819117
hRSV/A/USA/MD-JHSOM384/2024	EPI_ISL_19819119
hRSV/A/USA/MD-JHSOM387/2024	EPI_ISL_19819122
hRSV/A/USA/MD-JHSOM390/2024	EPI_ISL_19819125
hRSV/A/USA/MD-JHSOM392/2024	EPI_ISL_19819127

hRSV/A/USA/MD-JHSOM394/2024	EPI_ISL_19819129
hRSV/A/USA/MD-JHSOM397/2024	EPI_ISL_19819132
hRSV/A/USA/MD-JHSOM399/2024	EPI_ISL_19819133
hRSV/A/USA/MD-JHSOM402/2024	EPI_ISL_19819136
hRSV/A/USA/MD-JHSOM403/2024	EPI_ISL_19819137
hRSV/A/USA/MD-JHSOM404/2024	EPI_ISL_19819138
hRSV/A/USA/MD-JHSOM411/2024	EPI_ISL_19819145
hRSV/A/USA/MD-JHSOM415/2024	EPI_ISL_19819148
hRSV/A/USA/MD-JHSOM418/2024	EPI_ISL_19819150
hRSV/A/USA/MD-JHSOM426/2024	EPI_ISL_19819157
hRSV/A/USA/MD-JHSOM433/2024	EPI_ISL_19819164
hRSV/A/USA/MD-JHSOM435/2024	EPI_ISL_19819166
hRSV/A/USA/MD-JHSOM438/2024	EPI_ISL_19819168
hRSV/A/USA/MD-JHSOM447/2024	EPI_ISL_19819176
hRSV/A/USA/MD-JHSOM45/2024	EPI_ISL_19818855
hRSV/A/USA/MD-JHSOM46/2024	EPI_ISL_19818856
hRSV/A/USA/MD-JHSOM51/2024	EPI_ISL_19818860
hRSV/A/USA/MD-JHSOM54/2024	EPI_ISL_19818863
hRSV/A/USA/MD-JHSOM59/2024	EPI_ISL_19818867
hRSV/A/USA/MD-JHSOM61/2024	EPI_ISL_19818868
hRSV/A/USA/MD-JHSOM65/2024	EPI_ISL_19818872
hRSV/A/USA/MD-JHSOM67/2024	EPI_ISL_19818873
hRSV/A/USA/MD-JHSOM68/2024	EPI_ISL_19818874
hRSV/A/USA/MD-JHSOM72/2024	EPI_ISL_19818877
hRSV/A/USA/MD-JHSOM80/2024	EPI_ISL_19818885
hRSV/A/USA/MD-JHSOM82/2024	EPI_ISL_19818887
hRSV/A/USA/MD-JHSOM86/2024	EPI_ISL_19818890
hRSV/A/USA/MD-JHSOM89/2024	EPI_ISL_19818892
hRSV/A/USA/MD-JHSOM94/2024	EPI_ISL_19818896
hRSV/A/USA/MD-JHSOM96/2024	EPI_ISL_19818897
hRSV/B/USA/MD-JHRSV1001/2023	EPI_ISL_19884172
hRSV/B/USA/MD-JHRSV1002/2023	EPI_ISL_19884173
hRSV/B/USA/MD-JHRSV1004/2023	EPI_ISL_19884174
hRSV/B/USA/MD-JHRSV1005/2023	EPI_ISL_19884175
hRSV/B/USA/MD-JHRSV1006/2023	EPI_ISL_19884176
hRSV/B/USA/MD-JHRSV1008/2023	EPI_ISL_19884177
hRSV/B/USA/MD-JHRSV1009/2023	EPI_ISL_19884178
hRSV/B/USA/MD-JHRSV1010/2023	EPI_ISL_19884179
hRSV/B/USA/MD-JHRSV1012/2023	EPI_ISL_19884180
hRSV/B/USA/MD-JHRSV1013/2023	EPI_ISL_19884181
hRSV/B/USA/MD-JHRSV1014/2023	EPI_ISL_19884182

hRSV/B/USA/MD-JHRSV1015/2023	EPI_ISL_19884183
hRSV/B/USA/MD-JHRSV1016/2023	EPI_ISL_19884184
hRSV/B/USA/MD-JHRSV1017/2023	EPI_ISL_19884185
hRSV/B/USA/MD-JHRSV1018/2023	EPI_ISL_19884186
hRSV/B/USA/MD-JHRSV1019/2023	EPI_ISL_19884187
hRSV/B/USA/MD-JHRSV1022/2023	EPI_ISL_19884188
hRSV/B/USA/MD-JHRSV463/2019	EPI_ISL_19884061
hRSV/B/USA/MD-JHRSV476/2019	EPI_ISL_19884062
hRSV/B/USA/MD-JHRSV523/2019	EPI_ISL_19884063
hRSV/B/USA/MD-JHRSV543/2019	EPI_ISL_19884064
hRSV/B/USA/MD-JHRSV546/2019	EPI_ISL_19884065
hRSV/B/USA/MD-JHRSV556/2018	EPI_ISL_19884066
hRSV/B/USA/MD-JHRSV557/2018	EPI_ISL_19884067
hRSV/B/USA/MD-JHRSV561/2018	EPI_ISL_19884068
hRSV/B/USA/MD-JHRSV564/2018	EPI_ISL_19884069
hRSV/B/USA/MD-JHRSV566/2018	EPI_ISL_19884070
hRSV/B/USA/MD-JHRSV573/2018	EPI_ISL_19884071
hRSV/B/USA/MD-JHRSV575/2018	EPI_ISL_19884072
hRSV/B/USA/MD-JHRSV576/2018	EPI_ISL_19884073
hRSV/B/USA/MD-JHRSV586/2018	EPI_ISL_19884074
hRSV/B/USA/MD-JHRSV590/2018	EPI_ISL_19884075
hRSV/B/USA/MD-JHRSV591/2018	EPI_ISL_19884076
hRSV/B/USA/MD-JHRSV592/2018	EPI_ISL_19884077
hRSV/B/USA/MD-JHRSV598/2018	EPI_ISL_19884078
hRSV/B/USA/MD-JHRSV602/2018	EPI_ISL_19884079
hRSV/B/USA/MD-JHRSV608/2018	EPI_ISL_19884080
hRSV/B/USA/MD-JHRSV609/2018	EPI_ISL_19884081
hRSV/B/USA/MD-JHRSV610/2018	EPI_ISL_19884082
hRSV/B/USA/MD-JHRSV616/2018	EPI_ISL_19884083
hRSV/B/USA/MD-JHRSV617/2018	EPI_ISL_19884084
hRSV/B/USA/MD-JHRSV618/2018	EPI_ISL_19884085
hRSV/B/USA/MD-JHRSV619/2018	EPI_ISL_19884086
hRSV/B/USA/MD-JHRSV621/2018	EPI_ISL_19884087
hRSV/B/USA/MD-JHRSV623/2018	EPI_ISL_19884088
hRSV/B/USA/MD-JHRSV624/2018	EPI_ISL_19884089
hRSV/B/USA/MD-JHRSV625/2018	EPI_ISL_19884090
hRSV/B/USA/MD-JHRSV626/2018	EPI_ISL_19884091
hRSV/B/USA/MD-JHRSV627/2018	EPI_ISL_19884092
hRSV/B/USA/MD-JHRSV628/2018	EPI_ISL_19884093
hRSV/B/USA/MD-JHRSV629/2018	EPI_ISL_19884094
hRSV/B/USA/MD-JHRSV636/2018	EPI_ISL_19884095

hRSV/B/USA/MD-JHRSV637/2018	EPI_ISL_19884096
hRSV/B/USA/MD-JHRSV638/2018	EPI_ISL_19884097
hRSV/B/USA/MD-JHRSV639/2018	EPI_ISL_19884098
hRSV/B/USA/MD-JHRSV640/2018	EPI_ISL_19884099
hRSV/B/USA/MD-JHRSV641/2018	EPI_ISL_19884100
hRSV/B/USA/MD-JHRSV642/2018	EPI_ISL_19884101
hRSV/B/USA/MD-JHRSV645/2018	EPI_ISL_19884103
hRSV/B/USA/MD-JHRSV648/2018	EPI_ISL_19884104
hRSV/B/USA/MD-JHRSV649/2018	EPI_ISL_19884105
hRSV/B/USA/MD-JHRSV650/2018	EPI_ISL_19884106
hRSV/B/USA/MD-JHRSV657/2022	EPI_ISL_19884107
hRSV/B/USA/MD-JHRSV660/2022	EPI_ISL_19884108
hRSV/B/USA/MD-JHRSV662/2022	EPI_ISL_19884109
hRSV/B/USA/MD-JHRSV672/2022	EPI_ISL_19884110
hRSV/B/USA/MD-JHRSV686/2022	EPI_ISL_19884111
hRSV/B/USA/MD-JHRSV689/2022	EPI_ISL_19884112
hRSV/B/USA/MD-JHRSV690/2022	EPI_ISL_19884113
hRSV/B/USA/MD-JHRSV697/2022	EPI_ISL_19884114
hRSV/B/USA/MD-JHRSV700/2022	EPI_ISL_19884115
hRSV/B/USA/MD-JHRSV706/2022	EPI_ISL_19884116
hRSV/B/USA/MD-JHRSV709/2022	EPI_ISL_19884117
hRSV/B/USA/MD-JHRSV715/2022	EPI_ISL_19884118
hRSV/B/USA/MD-JHRSV717/2022	EPI_ISL_19884119
hRSV/B/USA/MD-JHRSV724/2022	EPI_ISL_19884120
hRSV/B/USA/MD-JHRSV749/2020	EPI_ISL_19884121
hRSV/B/USA/MD-JHRSV759/2020	EPI_ISL_19884122
hRSV/B/USA/MD-JHRSV765/2020	EPI_ISL_19884123
hRSV/B/USA/MD-JHRSV777/2020	EPI_ISL_19884124
hRSV/B/USA/MD-JHRSV797/2020	EPI_ISL_19884125
hRSV/B/USA/MD-JHRSV798/2020	EPI_ISL_19884126
hRSV/B/USA/MD-JHRSV814/2020	EPI_ISL_19884127
hRSV/B/USA/MD-JHRSV821/2020	EPI_ISL_19884128
hRSV/B/USA/MD-JHRSV822/2020	EPI_ISL_19884129
hRSV/B/USA/MD-JHRSV831/2020	EPI_ISL_19884130
hRSV/B/USA/MD-JHRSV929/2023	EPI_ISL_19884131
hRSV/B/USA/MD-JHRSV930/2023	EPI_ISL_19884132
hRSV/B/USA/MD-JHRSV931/2023	EPI_ISL_19884133
hRSV/B/USA/MD-JHRSV932/2023	EPI_ISL_19884134
hRSV/B/USA/MD-JHRSV933/2023	EPI_ISL_19884135
hRSV/B/USA/MD-JHRSV934/2023	EPI_ISL_19884136
hRSV/B/USA/MD-JHRSV935/2023	EPI_ISL_19884137

hRSV/B/USA/MD-JHRSV936/2023	EPI_ISL_19884138
hRSV/B/USA/MD-JHRSV937/2023	EPI_ISL_19884139
hRSV/B/USA/MD-JHRSV940/2023	EPI_ISL_19884140
hRSV/B/USA/MD-JHRSV941/2023	EPI_ISL_19884141
hRSV/B/USA/MD-JHRSV942/2023	EPI_ISL_19884142
hRSV/B/USA/MD-JHRSV945/2023	EPI_ISL_19884143
hRSV/B/USA/MD-JHRSV946/2023	EPI_ISL_19884144
hRSV/B/USA/MD-JHRSV948/2023	EPI_ISL_19884145
hRSV/B/USA/MD-JHRSV950/2023	EPI_ISL_19884146
hRSV/B/USA/MD-JHRSV952/2023	EPI_ISL_19884147
hRSV/B/USA/MD-JHRSV953/2023	EPI_ISL_19884148
hRSV/B/USA/MD-JHRSV954/2023	EPI_ISL_19884149
hRSV/B/USA/MD-JHRSV958/2023	EPI_ISL_19884150
hRSV/B/USA/MD-JHRSV959/2023	EPI_ISL_19884151
hRSV/B/USA/MD-JHRSV961/2023	EPI_ISL_19884152
hRSV/B/USA/MD-JHRSV964/2023	EPI_ISL_19884153
hRSV/B/USA/MD-JHRSV966/2023	EPI_ISL_19884154
hRSV/B/USA/MD-JHRSV967/2023	EPI_ISL_19884155
hRSV/B/USA/MD-JHRSV968/2023	EPI_ISL_19884156
hRSV/B/USA/MD-JHRSV969/2023	EPI_ISL_19884157
hRSV/B/USA/MD-JHRSV971/2023	EPI_ISL_19884158
hRSV/B/USA/MD-JHRSV972/2023	EPI_ISL_19884159
hRSV/B/USA/MD-JHRSV973/2023	EPI_ISL_19884160
hRSV/B/USA/MD-JHRSV974/2023	EPI_ISL_19884161
hRSV/B/USA/MD-JHRSV980/2023	EPI_ISL_19884162
hRSV/B/USA/MD-JHRSV983/2023	EPI_ISL_19884163
hRSV/B/USA/MD-JHRSV989/2023	EPI_ISL_19884164
hRSV/B/USA/MD-JHRSV990/2023	EPI_ISL_19884165
hRSV/B/USA/MD-JHRSV991/2023	EPI_ISL_19884166
hRSV/B/USA/MD-JHRSV993/2023	EPI_ISL_19884167
hRSV/B/USA/MD-JHRSV994/2023	EPI_ISL_19884168
hRSV/B/USA/MD-JHRSV996/2023	EPI_ISL_19884169
hRSV/B/USA/MD-JHRSV997/2023	EPI_ISL_19884170
hRSV/B/USA/MD-JHRSV999/2023	EPI_ISL_19884171
hRSV/B/USA/MD-JHSOM122/2024	EPI_ISL_19818917
hRSV/B/USA/MD-JHSOM175/2024	EPI_ISL_19818960
hRSV/B/USA/MD-JHSOM202/2024	EPI_ISL_19818983
hRSV/B/USA/MD-JHSOM243/2024	EPI_ISL_19819011
hRSV/B/USA/MD-JHSOM264/2024	EPI_ISL_19819024
hRSV/B/USA/MD-JHSOM326/2024	EPI_ISL_19819071
hRSV/B/USA/MD-JHSOM337/2024	EPI_ISL_19819080

hRSV/B/USA/MD-JHSOM344/2024	EPI_ISL_19819087
hRSV/B/USA/MD-JHSOM43/2024	EPI_ISL_19818853
hRSV/B/USA/MD-JHSOM437/2024	EPI_ISL_19819167
hRSV/B/USA/MD-JHSOM451/2024	EPI_ISL_19819179
hRSV/B/USA/MD-JHSOM457/2024	EPI_ISL_19819185
hRSV/B/USA/MD-JHSOM459/2024	EPI_ISL_19819187
hRSV/B/USA/MD-JHSOM47/2024	EPI_ISL_19818857
hRSV/B/USA/MD-JHSOM52/2024	EPI_ISL_19818861
hRSV/B/USA/MD-JHSOM77/2024	EPI_ISL_19818882
hRSV/B/USA/MD-JHSOM79/2024	EPI_ISL_19818884
